# Synergistic Ni‐Co Metal Nodes in a Conjugated MOF‐Modified Separator for High‐Performance Lithium‐Sulfur Batteries

**DOI:** 10.1002/advs.202513282

**Published:** 2025-09-09

**Authors:** Yuanhang Xu, Yuxuan Jiang, Xiang Yu, Yijing Gu, Mohsen Shakouri, Rongmei Zhu, Huan Pang

**Affiliations:** ^1^ School of Chemistry and Chemical Engineering Yangzhou University Yangzhou Jiangsu 225009 P. R. China; ^2^ School of Chemistry and Chemical Engineering Chongqing University of Science and Technology Chongqing 401331 P. R. China; ^3^ School of Environmental Science Nanjing Xiaozhuang University Nanjing Jiangsu 211171 P. R. China; ^4^ Canadian Light Source University of Saskatchewan Saskatoon Saskatchewan S7N 2V3 Canada

**Keywords:** conjugated MOF, Li‐S batteries, separators, synergistic effect

## Abstract

Lithium‐sulfur batteries (LSBs) hold great potential as next‐generation energy storage systems due to their high theoretical energy density and relatively low cost. However, their practical application is hindered by issues such as the shuttle phenomenon caused by soluble lithium polysulfides (LiPSs), slow redox reaction rates, and unsatisfactory cycling stability. In this study, novel conjugated metal–organic frameworks, M_x_M″_3‐x_(HHTP)_2_ (M, M″ = Ni^2+^, Co^2+^, Cu^2+^) is reported, as a functional coating on polypropylene (PP) separators. Leveraging the in‐plane d‐π conjugation, high porosity, and rich redox‐active sites, the M_x_M'_3‐x_(HHTP)_2_‐modified separators effectively suppress LiPSs’ shuttling, facilitate Li^+^ transport, and accelerate LiPSs’ redox conversion. Among the series, the bimetallic Ni_1.35_Co_1.65_(HHTP)_2_/PP separator demonstrates superior electrochemical performance, owing to the synergistic interaction between Ni and Co metal centers. This synergism results in an optimized pore structure, enhances conductivity, and stronger polysulfide affinity compare to monometallic analogues. Consequently, the cell employing Ni_1.3_5Co_1.65_(HHTP)_2_/PP provides an initial discharge capacity of 1148 mAh g^−1^ at 0.1 C and the capacity decay rate is 0.08% after 500 cycles at 1C. This work presents a scalable and environmentally friendly strategy for constructing multifunctional separators to fully realize the potential of high‐energy‐density LSBs.

## Graphical abstract

In this work, the employment of cMOFs is reported as modified materials for LSB separators. It is proved that cMOF with in‐plane d‐π conjugation is a good barrier layer for constructing high‐performance LSBs. Through a series of in situ and non in situ electrochemical examinations, LSB assembled with Ni_1.35_Co_1.65_(HHTP)_2_ separator exhibits excellent electrochemical performance.

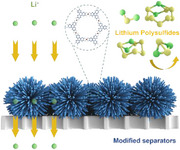

## 1 Introduction

As the demand for renewable energy continues to rise, high energy density batteries have garnered significant attention from both academia and industry.^[^ ^1^ ^]^ Of course, there are still many problems in improving battery performance, such as the theoretical capacity limitation of materials, the influence of volume change and some side reactions on performance during charging and discharging, the safety problems of high energy density batteries, the reduction of performance at low temperature and cost problems.^[^ ^2^ ^]^ For the existence of these problems, many researchers have proposed their own solutions. For example, Liu et al. studied the influence of temperature on lithium‐ion batteries (LIBs), and systematically introduced the factors of performance degradation of LIBs at low temperatures.^[^ ^3^ ^]^ Chen et al. provided an idea for the problem of sodium dendrite growth in sodium metal batteries;^[^ ^4^ ^]^ Wang et al. designed four typical internal short‐circuit experiments, analyzed the internal short‐circuit process of the battery, and provided new ideas for the development of high‐safety batteries.^[^ ^5^ ^]^ Among these, lithium‐sulfur batteries (LSBs) have gained considerable attention because of their impressive theoretical specific capacity (1675 mAh g^−1^) and energy density (2600 Wh kg^−1^).^[^ ^6^ ^]^ Thanks to the progress of sulfur carrier materials, the specific capacity and cycle stability of LSBs cathode have been significantly improved.^[^ ^7^ ^]^ Unfortunately, the rapid capacity decay of high‐sulfur cathodes caused by the shuttle of polysulfide (Li_2_S_n_; 2 ≤ n ≤ 8) intermediates is still a bottleneck restricting the development of high‐energy density LSBs.^[^ ^8^ ^]^ Therefore, suppressing the shuttle effect and enhancing active material utilization are essential for developing high‐performance LSBs.^[^ ^9^ ^]^ An effective way to solve these problems is to use different host materials (e.g., carbon materials, conductive polymers,^[^ ^10^ ^]^ Prussian blue,^[^ ^11^ ^]^ etc.) to modify the S cathode by encapsulation or layer‐by‐layer assembly.^[^ ^12^ ^]^

It has been shown in recent studies that the separator plays a vital role in overcoming the critical issues of LSBs, particularly in addressing issues like the shuttle effect, dendritic deposition, interfacial robustness, and system safety.^[^ ^13^ ^]^ For example, Manthiram and Su inhibit polysulfide shuttle through microporous carbon paper interlayers to improve the utilization of S cathode.^[^ ^14^ ^]^ Since then, researchers have used carbon to modify the separator to inhibit the shuttle of Li_2_S_n_. Nevertheless, owing to the limited interaction between nonpolar carbon frameworks and polar Li_2_S_n_, the shuttle phenomenon remains pronounced during long‐term operation.^[^ ^15^ ^]^ Therefore, different polar materials are employed to modify the separator, such as carbon materials with polar groups,^[^ ^16^ ^]^ metal oxides,^[^ ^17^ ^]^ metal sulfides,^[^ ^18^ ^]^ and black phosphorus.^[^ ^19^ ^]^ Although such modified separators effectively restrict the migration of Li_2_S_n_, they inevitably compromise the transport of Li^+^ ions. Hence, the fabrication of separators capable of effectively blocking polysulfide migration while ensuring excellent Li⁺ conductivity is still difficult to achieve, especially via environmentally benign and economical methods.^[^ ^20^ ^]^ Metal‐organic frameworks (MOFs) have the characteristics of high porosity, adjustable pore parameters and high affinity to guest molecules, and show great application potential in the fields of gas storage and separation,^[^ ^21^ ^]^ catalysis,^[^ ^22^ ^]^ sensing,^[^ ^23^ ^]^ energy conversion and storage.^[^ ^24^ ^]^ Typically, 2D cMOFs are constructed from metal centers and π‐conjugated organic ligands.^[^ ^25^ ^]^ Enhanced in‐plane d‐π conjugation within cMOFs promotes charge transport and leads to improved conductivity. Meanwhile, they possess the inherent benefits of conventional MOFs, including compositional tunability, customizable pore structures, and well‐defined active sites.^[^ ^26^ ^]^ Importantly, cMOFs combine high electrical conductivity with abundant active sites, which make them have potential application prospects in inhibiting the shuttle effect.

In this work, we reported the use of cMOF M_x_M″_3‐x_(HHTP)_2_ (M, M″ = Ni^2+^, Co^2+^ and Cu^2+^, HHTP = 2,3,6,7,10,11‐triphenylenehexol) as a modified material for LSB separators. It is proved that the cMOF with in‐plane d‐π conjugation is a good barrier layer for the construction of high‐performance LSBs. Its uniform pore size (∼ 1.8 nm) can effectively inhibit the shuttle of long‐chain polysulfides. Due to the unique structural design, the LSB with Ni_1.35_Co_1.65_ (HHTP)_2_ coated polypropylene (PP) as the separator can provide excellent specific capacitance even at high current, and exhibits excellent capacity retention of 58% after 500 cycles at 1.0 C.

## 2 Results and Discussion

**Figure 1:**
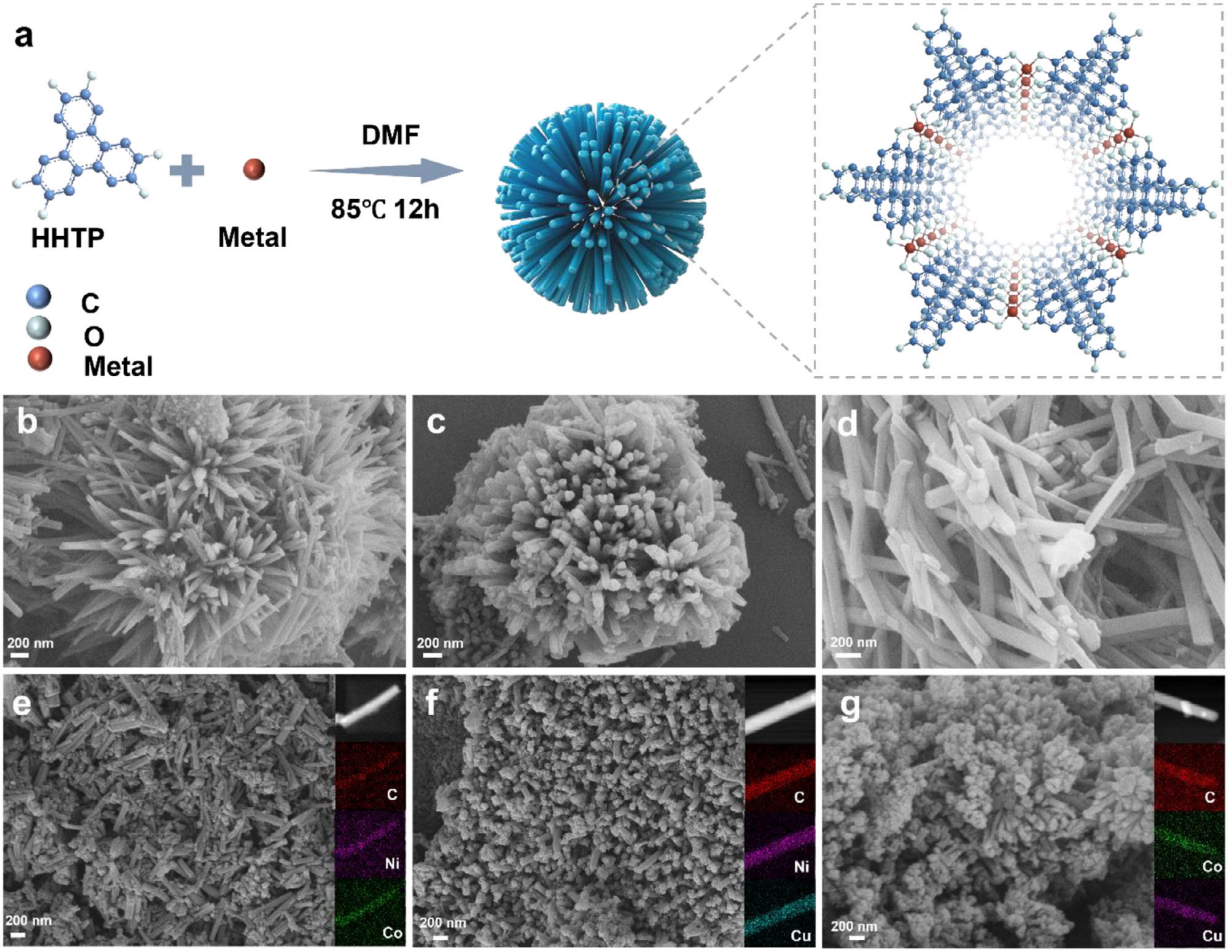
a) The synthesis diagram of M_x_M'_3‐x_(HHTP)_2_; SEM images of b) Ni_3_(HHTP)_2_ nanorods, c) Co_3_(HHTP)_2_ nanorods, d) Cu_3_(HHTP)_2_ nanorods, e) Ni_1.35_Co_1.65_ (HHTP)_2_ nanorods, f) Ni_0.89_Cu_2.11_(HHTP)_2_ nanorods, g) Co_0.92_Cu_2.08_(HHTP)_2_ nanorods, the insets are their elemental mappings.

The synthesis route for M_x_M″_3‐x_(HHTP)_2_ is illustrated in **Figure** **1**a. M_x_M″_3‐x_(HHTP)_2_ was prepared using a one‐step solvothermal method. The assembly of metal ions and π‐conjugated ligand HHTP produced a new class of 2D layered MOF. Compared with the traditional MOFs, this type of MOF is very different due to its high electronic conductivity and unique structural characteristics. Structurally, the HHTP ligand coordinates with M^2+^ ions in the ab plane, forming a hexagonal layer (Figure S1a, Supporting Information). Along the c‐axis, M_x_M″_3‐x_(HHTP)_2_ displays an AA stacking arrangement with π‐π interactions, as shown in Figure S1b (Supporting Information). Due to the strong charge delocalization between M^2+^ and the ligand, M_x_M″_3‐x_(HHTP)_2_ exhibits good electronic conductivity. Taking Cu_3_(HHTP)_2_ as an example, it is found that Cu_3_(HHTP)_2_ is one of the best cMOFs reported so far, and its conductivity is 2.1×10^−1^ S cm^−1^, which is much larger than that of traditional MOFs. It is worth noting that the hydroxyl (‐OH) groups in HHTP can adsorb lithium polysulfides (LiPSs) through hydrogen bonding, and may also coordinate with Li⁺ ions via phenolate oxygen atoms under suitable conditions.^[^ ^27^ ^]^ This adsorption can inhibit the dissolution of Li_2_S_n_ in the electrolyte, thereby reducing the shuttle effect. In addition, HHTP can inhibit the redox reaction of Li_2_S_n_ through its antioxidant properties. This helps to improve the recyclability of the sulfur cathode and avoid capacity decay caused by Li_2_S_n_ reduction and oxidation in LSBs.

The metal content and molar ratio in the bimetallic MOFs were verified by inductively coupled plasma emission spectroscopy (ICP) analysis (Table S1, Supporting Information). The morphology of the synthesized M_x_M″_3‐x_(HHTP)_2_ is displayed in Figure 1b–g. The inset in the figure are their respective elemental mappings. It can be seen that the metal elements are evenly distributed in M_x_M″_3‐x_(HHTP)_2_. Transmission electron microscopy (TEM) images (Figure S2, Supporting Information) of M_x_M″_3‐x_(HHTP)_2_ also show nanorod morphology, which is similar to the SEM results. The crystal structure of M_x_M″_3‐x_(HHTP)_2_ was further studied using powder X‐ray diffraction (PXRD). The primary diffraction peaks were observed at approximately 2θ = 9.5°, 12.5°, and 28°, which correspond to the (200), (210), and (001) crystal planes, respectively, as illustrated in Figure S3 (Supporting Information). Moreover, the introduction of Co in the M_x_M″_3‐x_(HHTP)_2_ framework leads to a significant decrease in the intensity of the characteristic XRD peaks. This attenuation can be attributed to the local lattice distortion and structural disorder caused by the substitution of Co^2+^ for Ni^2+^/Cu^2+^, which destroys the long‐range crystallinity of the MOF layer.^[^ ^28^ ^]^ In addition, the potential decrease of grain size and the increase of microstrain may further lead to the broadening and weakening of diffraction peaks. These observations imply that bimetallic substitution affects the stacking behavior and ordering of the cMOF layers, particularly along the c‐axis direction. A non‐hk0 reflection is observed at 2θ ≈ 28°, corresponding to the (001) plane, which arises from periodic stacking along the c‐axis. This peak is consistent with an AA‐stacking configuration, suggesting that the layers are aligned without lateral displacement. The AA arrangement is likely stabilized by weak interlayer interactions between adjacent metal centers. These findings confirm that the synthesized M_x_M″_3‐x_(HHTP)_2_ crystals have high crystallinity. Fourier transform infrared spectroscopy revealed strong absorption peaks at 1214 cm^−1^ and 1500 cm^−1^ for M_x_M″_3‐x_(HHTP)_2_, corresponding to the stretching vibrations of C─O and C═O functional groups, respectively (Figure S4, Supporting Information). Notably, all samples incorporating different metal centers exhibited consistent peak positions, indicating that the organic ligand environment remained unchanged. This consistency confirms the successful synthesis of the M_x_M″_3‐x_(HHTP)_2_ framework across all compositions. To further confirm the pore size characteristics of the cMOF layer, N_2_ adsorption‐desorption measurements were performed on the synthesized Ni_1.35_Co_1.65_(HHTP)_2_ and Ni_0.89_Cu_2.11_(HHTP)_2_. As shown in Figures S5 and S6 (Supporting Information), the isotherm exhibits a typical type II profile, indicating the presence of abundant mesopores. The Bruno‐Emmett‐Teller (BET) specific surface area of Ni_1.35_Co_1.65_(HHTP)_2_ is 196.26 m^2^ g^−1^, and the average pore size is about 11.3 nm. The BET specific surface area of b is 146.55 m^2^ g^−1^, and the average pore size is 7.3 nm. These results are consistent with the expected porous skeleton structure, indicating that the cMOF layer provides sufficient accessible channels for Li selective transport while effectively inhibiting the migration of LiPSs.

**Figure 2:**
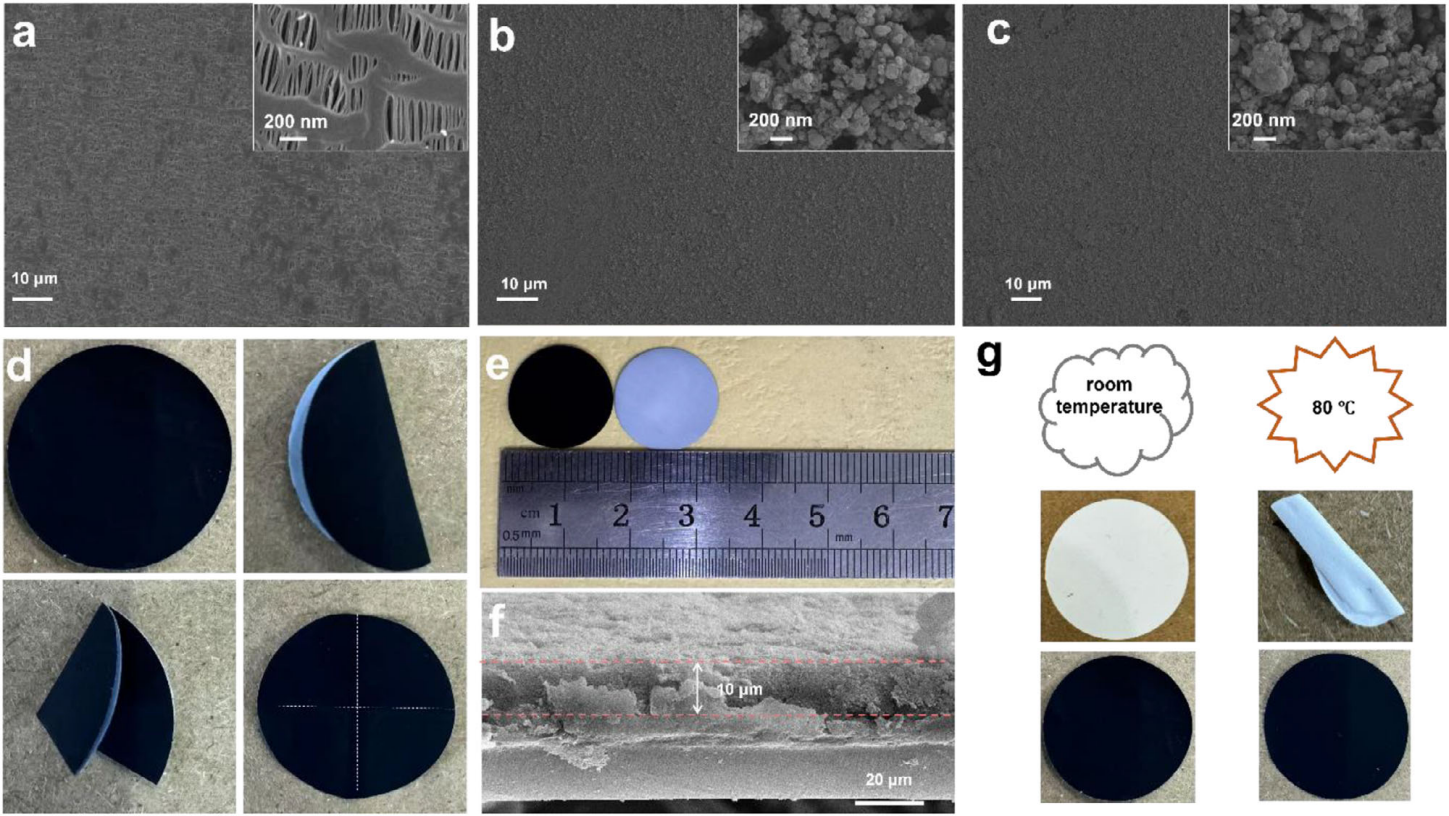
SEM images of a) original PP, b) Ni_1.35_Co_1.65_(HHTP)_2_/PP and c) Ni_0.89_Cu_2.11_(HHTP)_2_/PP. d) Digital images of M_x_M″_3‐x_(HHTP)_2_/PP separator after one, two folding and final restoration. e) Digital images of M_x_M″_3‐x_(HHTP)_2_/PP front and back and size. f) Cross‐section SEM image of the M_x_M″_3‐x_(HHTP)_2_/PP. g) Thermal stability test of M_x_M″_3‐x_(HHTP)_2_/PP and PP at room temperature and 80 °C.

The primary objective of the modified separator developed in this study is to prevent the shuttle effect of Li_2_S_n_, thereby enhancing the electrochemical performance of LSBs, the modified separator should be able to prevent the shuttle of Li_2_S_n_ effectively and electrocatalytically convert the captured Li_2_S_n_, while providing sufficient space for the free transport of salts, solvents and Li^+^ on the separator. The effects of the structure and morphology of the separator on the transport properties of Li_2_S_n_ and Li^+^ were studied. The SEM images of the pristine PP separator and the modified Ni_1.35_Co_1.65_(HHTP)_2_/PP and Ni_0.89_Cu_2.11_(HHTP)_2_/PP separators are shown in **Figure** **2**a–c, with corresponding magnified views provided in the insets. The PP separator features a relatively smooth surface combined with a nanoporous structure (≈100 nm pore diameter), which promotes fast lithium‐ion transport. However, its porous nature also offers little resistance to the diffusion of LiPSs, allowing them to migrate toward the anode and induce the shuttle effect. The SEM image of the M_x_M″_3‐x_(HHTP)_2_ coated separator clearly shows that the separator is completely coated with M_x_M″_3‐x_(HHTP)_2_. This coating is designed to provide the pore structure and function to block Li_2_S_n_. The uniform pore size (≈1.8 nm) in the MOF is beneficial to the rapid transport of Li^+^ (≈0.076 nm) and prevents the diffusion of LiPSs (≈1.5 nm). As shown in Figure 2d, the M_x_M″_3‐x_(HHTP)_2_‐coated separator retains its original shape after repeated bending, demonstrating strong adhesion between the functional layer and the PP substrate, as well as excellent mechanical robustness and flexibility. The folded separator exhibits extraordinary toughness and ensures close contact with the sulfur cathode surface, which is crucial for maintaining interface stability during the cycle. Figure 2e displays the front and back optical images of the modified separator, confirming its uniform coverage and dimensions. Furthermore, the cross‐sectional SEM image (Figure 2f) reveals that the M_x_M″_3‐x_(HHTP)_2_ coating is evenly distributed on the PP separator, with a uniform thickness of approximately 10 µm. Thermal stability is another crucial parameter for ensuring battery safety. Unlike conventional commercial separators, the M_x_M'_3‐x_(HHTP)_2_‐coated separator exhibits excellent thermal resistance and shows no observable shrinkage when heated to 80 °C (Figure 2g). This superior heat tolerance effectively minimizes the risk of internal short circuits under elevated temperatures, thereby enhancing the operational safety of LSBs.

**Figure 3:**
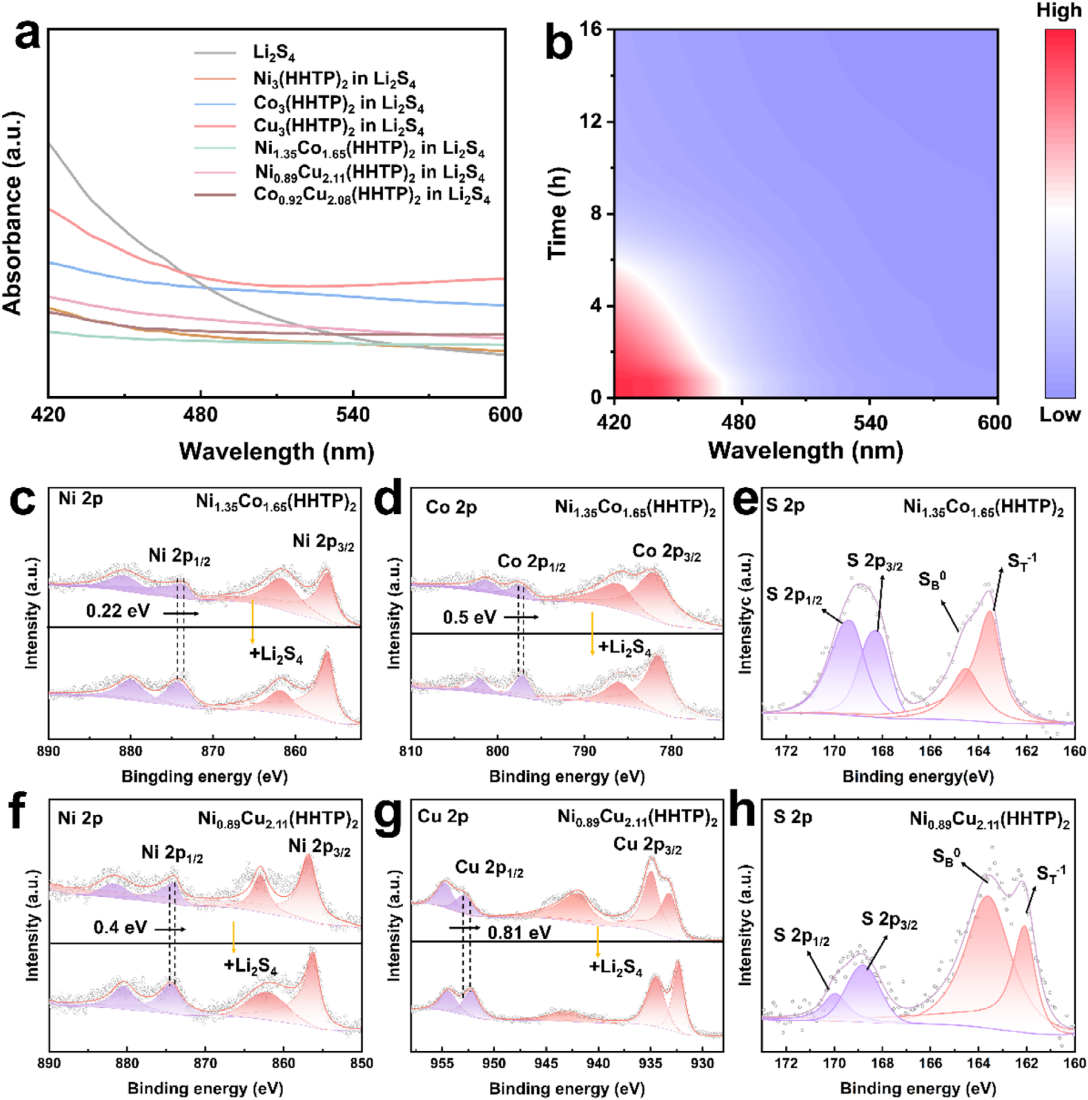
a) UV–vis absorption spectra of Li_2_S_4_ solution before and after adding M_x_M'_3‐x_(HHTP)_2_ for 24 h; b) In situ UV/Vis spectra of Ni_1.35_Co_1.65_(HHTP)_2_/Li_2_S_4_; High‐resolution XPS spectra of c) Ni, d) Co and e) S in Ni_1.35_Co_1.65_(HHTP)_2_ and Ni_1.35_Co_1.65_(HHTP)_2_/Li_2_S_4_. High‐resolution XPS spectra of f) Ni, g) Cu and h) S in Ni_0.89_Cu_2.11_(HHTP)_2_ and Ni_0.89_Cu_2.11_ (HHTP)_2_/Li_2_S_4_.

The structure and morphology analysis confirmed the successful synthesis of M_x_M″_3‐x_(HHTP)_2_ materials. In order to further evaluate its practical effect in inhibiting shuttle effect and improving battery performance, we focus on the analysis and comparison of Ni_1.35_Co_1.65_(HHTP)_2_ and Ni_0.89_Cu_2.11_(HHTP)_2_ MOFs. To evaluate the LiPSs adsorption capability of the samples, static adsorption experiments were conducted by immersing equal masses of M_x_M″_3‐x_(HHTP)_2_ into Li_2_S_4_ solutions. As shown in Figure S7 (Supporting Information), the solution treated with monometallic M_x_M'_3‐x_(HHTP)_2_ gradually changed from dark brown to nearly transparent after 24 hours, indicating substantial Li_2_S_4_ adsorption. Notably, the bimetallic HHTP sample achieved similar decolorization within just 12 hours, suggesting a synergistic effect that enhances the adsorption rate. The supernatants were collected under inert conditions and analyzed via ultraviolet‐visible spectroscopy (UV‐Vis) spectroscopy (**Figure** **3**a). In the presence of bimetallic samples, there is an obvious absorption peak near 420 nm, which is the characteristic peak of Li_2_S_4_, confirming the excellent affinity and rapid adsorption kinetics for LiPSs. In addition, to investigate the dynamic process of sample adsorption of Li_2_S_4_, we carried out in situ UV–vis test on diluted Li_2_S_4_ solution containing Ni_1.35_Co_1.65_(HHTP)_2_ sample. In situ UV–vis absorption spectroscopy showed that at the beginning of the experiment (about 0–3 h), an obvious absorption peak appeared at 420 nm, and the initial yellow solution of Ni_1.35_Cu_1.65_(HHTP)_2_ became a clear solution with almost no Li_2_S_4_ peak at 420 nm after standing for 6 h (Figure 3b; Figure S8, Supporting Information). This is because HHTP provides abundant polyphenol hydroxyl sites, which is conducive to hydrogen bonding or π‐π interaction with Li_2_S_4_, while the NiCo bimetallic center enhances the selective adsorption of polysulfides through strong polar interaction, and the synergistic effect enables it to quickly and efficiently capture Li_2_S_4_.

To visually illustrate the effectiveness of the prepared functional separators in inhibiting LiPSs diffusion, H‐type diffusion cells were assembled using different separators to simulate LiPSs migration during LSBs operation. Under identical conditions, PP, Ni_1.35_Co_1.65_(HHTP)_2_/PP, and Ni_0.89_Cu_2.11_(HHTP)_2_/PP separators were evaluated for their ability to block LiPSs permeation over time (Figure S9, Supporting Information). For the pristine PP separator, the yellow‐colored LiPSs solution visibly diffused to the opposite chamber within just 3 hours, indicating severe Li_2_S_n_ crossover. In contrast, both Ni_1.35_Co_1.65_(HHTP)_2_/PP and Ni_0.89_Cu_2.11_(HHTP)_2_/PP significantly suppressed the diffusion of LiPSs, with only a faint yellow coloration observed even after 12 hours. These observations confirm the strong blocking capability of the M_x_M'_3‐x_(HHTP)_2_‐coated separators, effectively mitigating the shuttle effect and thus contributing to enhanced cycling stability and prolonged service life of LSBs. To elucidate the underlying mechanism responsible for the enhanced Li_2_S_n_ confinement, X‐ray photoelectron spectroscopy (XPS) analyses were conducted on Ni_1.35_Co_1.65_(HHTP)_2_ and Ni_0.89_Cu_2.11_(HHTP)_2_ before and after Li_2_S_4_ adsorption. In the Ni_1.35_Co_1.65_(HHTP)_2_ sample, the binding energies of Ni 2p_1/2_ and Co 2p_1/2_ decreased by 0.22 eV and 0.50 eV, respectively (Figure 3c,d), indicating chemical interactions with LiPSs. These shifts suggest that both Ni and Co sites are actively involved in LiPSs anchoring, benefitting from synergistic orbital interactions that facilitate electron redistribution and redox catalysis. In comparison, the Ni_0.89_Cu_2.11_(HHTP)_2_ sample exhibited binding energy shifts of 0.40 eV (Ni 2p_1/2_) and 0.81 eV (Cu 2p_1/2_) (Figure 3f,g), indicating some degree of interaction with LiPSs. We believe that the binding energy offset of Cu 2p_1/2_ is significantly larger than that of Ni 2p_1/2_ and Co 2p_1/2_, because the electronic structure of Cu 2p_1/2_ is more susceptible to the influence of coordination environment. In addition, the Jahn‐Teller effect of Cu 2p_1/2_ leads to the change of the coordination structure of Cu with O and S atoms.^[^ ^29^ ^]^ When the S atoms of LiPSs are close, it is easy to cause local electronic structure reconstruction, which further enlarges the binding energy offset of Cu^2+^, while Ni^2+^ and Co^2+^ are more symmetrical and more stable. Additionally, the S 2p spectra (Figure 3e,h) for both samples revealed characteristic peaks for terminal sulfur (ST^−1^) and bridging sulfur (SB^0^) at ≈163 eV and ≈164 eV, as well as signals corresponding to S 2p_3/2_ and S 2p_1/2_ at ≈168–169 eV. These findings confirm the effective chemisorption of LiPSs, particularly in the Ni‐Co bimetallic system, which exhibits a more favorable electronic environment for suppressing the shuttle effect.

**Figure 4:**
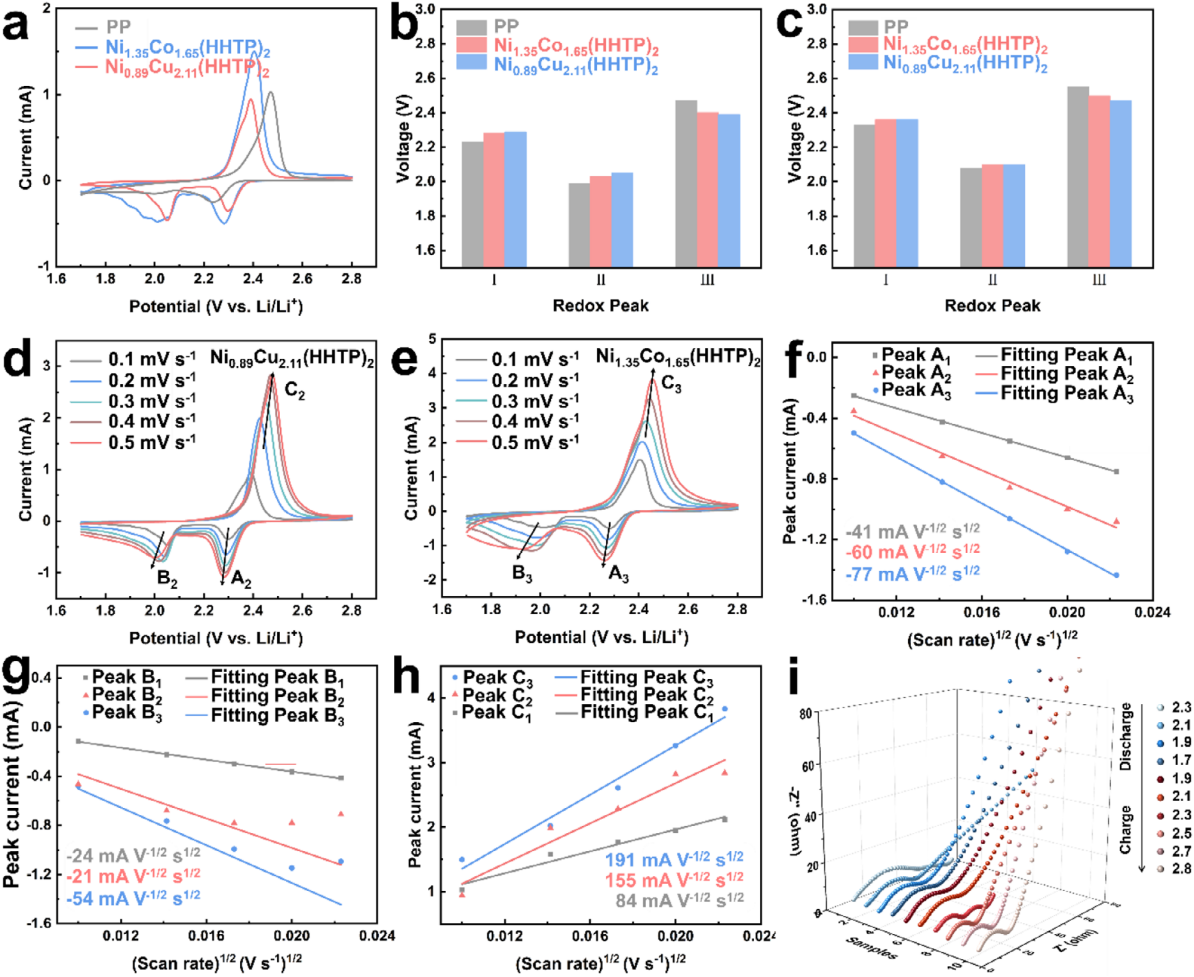
CV profiles a), peak voltages b), and onset potential c), of the LSBs based on PP, Ni_1.35_Co_1.65_(HHTP)_2_/PP, and Ni_0.89_Cu_2.11_(HHTP)_2_/PP. The CV curves of LSBs with different separators at different scanning rates, d) Ni_0.89_Cu_2.11_(HHTP)_2_/PP and e) Ni_1.35_Co_1.65_(HHTP)_2_/PP. The plots of peak current versus the square root of the scan rate (v^1/2^) for f) the transformation process from S8 to Li_2_S_x_, g) the transformation process from Li_2_S_x_ to Li_2_S and h) the transformation process from Li_2_S to S_8_; i) In situ EIS spectra of the battery with Ni_1.35_Co_1.65_(HHTP)_2_ modified separator.

Cyclic voltammetry (CV) tests were performed to assess the effectiveness of M_x_M″_3‐x_(HHTP)_2_ as a separator modification material for LSBs, using cells with pristine PP, Ni_1.35_Co_1.65_(HHTP)_2_/PP, and Ni_0.89_Cu_2.11_(HHTP)_2_/PP separators. The measurements were conducted within a voltage range of 1.7‐2.8 V, with a scan rate of 0.1 mV s^−1^ (**Figure** **4**a). The cells using modified separators exhibited two distinct cathodic peaks: 2.29 V and 2.04 V for Ni_1.35_Co_1.65_(HHTP)_2_, and 2.27 V and 2.01 V for Ni_0.89_Cu_2.11_(HHTP)_2_. These peaks correspond to the two‐step reduction of sulfur: first, the conversion of solid S_8_ into soluble Li_2_S_x_ species, followed by the further reduction of Li_2_S_x_ to insoluble Li_2_S_2_/Li_2_S. The anodic peak around 2.4 V represents the reverse oxidation of short‐chain lithium sulfides. Compared with the unmodified PP separator, the M_x_M″_3‐x_(HHTP)_2_‐modified separators not only show higher peak currents but also exhibit cathodic peak shifts toward more positive potentials, indicating reduced polarization and improved redox kinetics (Figure 4b). These improvements highlight the electrocatalytic activity of the cMOF coating and its ability to facilitate Li_2_S_n_ conversion. Moreover, the narrowed potential gap between the redox peaks indicates improved Li^+^ diffusion and increased charge‐transfer efficiency (Figure 4c). This is particularly beneficial for maintaining battery stability under high current rates, where ion transport is crucial to the overall electrochemical performance.

The lithium ions diffusion coefficient in LSBs was calculated from CV measurements taken at scan rates between 0.1 and 0.5 mV s^−1^. The curves exhibited the typical redox signals of LSBs (Figure 4d,e; Figure S10, Supporting Information). As the scanning rate increases, the oxidation peak potential of the sample shifts positively and the reduction peak potential shifts negatively. This is caused by the fact that, with an increase in scanning speed, electron transport inside the cell is faster than ion transport, resulting in increased polarization.

According to the Randles‐Sevcik equation:

(1) $$I_{p}=2.69\times 10^{5}n^{3/2}AD_{Li+}^{1/2}v^{1/2}C_{Li+}$$

In which, Ip, D_Li+_ and ν represent the peak current, lithium‐ion diffusion coefficient and scan rate, respectively, while n, A and C_Li+_ represent the number of electrons involved in the electrochemical reaction, the effective electrode area and the lithium‐ion concentration in the electrolyte. The lithium‐ion diffusion rate can be observed by the linear correlation between the peak current and the square root of the scan rate.

The diffusion coefficient of lithium ions (Figure S11, Supporting Information) was calculated according to the RandlesSevcik equation. The results show that the D_Li+_ of Ni_1.35_Co_1.65_(HHTP)_2_ is higher than that of Ni_0.89_Cu_2.11_(HHTP)_2_ at the three peaks of A, B, and C representing the redox process. This is consistent with the above analysis results of CV, and further proves that the metal synergy between Ni‐Co contributes to the conversion between sulfur and LiPS.

In addition, the Li^+^ diffusion kinetics were analyzed by examining the linear relationship between peak current (I_p_) and the square root of the scan rate (ν^1/2^), as shown in Figure 4f–h. The calculated slope values (K) of the fitted lines reveal that cells with Ni_1.35_Co_1.65_(HHTP)_2_ separators exhibit steeper slopes (Peak A_3_: ‐77, B_3_: ‐54, C_3_: 191 mA V^−1/2^ s^1/2^) compared to Ni_0.89_Cu_2.11_(HHTP)_2_ (A_2_: ‐60, B_2_: ‐21, C_2_: 155 mA V^−1/2^ s^1/2^) and pristine PP (A_1_: ‐41, B_1_: ‐24, C_1_: 84 mA V^−1/2^ s^1/2^). These steeper slopes indicate enhanced Li^+^ diffusion rates, which are beneficial for reducing concentration polarization and improving reaction kinetics. The improved performance of the bimetallic MOF systems is attributed to their continuous catalytic ability to promote LiPSs conversion throughout the redox process. This not only mitigates the shuttle effect but also enhances sulfur utilization. These benefits can be ascribed to the extended in‐plane d‐π conjugation and high electronic conductivity of the cMOF, which enable fast charge transport and efficient catalytic activity across all stages of the battery cycle.

Electrochemical impedance spectroscopy (EIS) was performed to further assess the influence of M_x_M″_3‐x_(HHTP)_2_ on LiPSs conversion dynamics. Figure S12 (Supporting Information) shows that the Nyquist plots reveal the key resistive elements: solution resistance (R_0_), cathode–electrolyte interfacial resistance (R_s_), charge‐transfer resistance (R_ct_), and Warburg impedance at low frequencies. Compared with the unmodified PP separator, the M_x_M″_3‐x_(HHTP)_2_‐modified separators, particularly Ni_1.35_Co_1.65_(HHTP)_2_/PP, exhibit significantly lower R_0_, R_s_, and R_ct_ values, indicating improved interfacial contact and enhanced charge‐transfer kinetics. The reduced semicircle diameter in the high‐frequency region further confirms the excellent conductivity of the Ni_1.35_Co_1.65_(HHTP)_2_ coating. In order to fully understand that the HHTP modified separator can effectively inhibit the polysulfide shuttle and enhance interfacial reaction kinetics, we performed an in situ EIS test on the Ni_1.35_Co_1.65_(HHTP)_2_/PP sample. As shown in Figure 4i, the moderate increase in R_ct_ during discharge implies that the insulating Li_2_S is more evenly deposited and does not severely hinder charge transfer, which benefits from the effective polysulfide anchoring capability of cMOFs. During charging, the rapid decrease of R_ct_ from 2.1 V to 2.3 V suggests that the anchored polysulfides can be efficiently reactivated and transported back to the cathode side, promoting the reversible conversion of Li_2_S. The stable R_s_ values further confirm that the introduction of cMOFs does not hinder ion transport but instead helps maintain continuous pathways for both ions and electrons. As shown in the Figures S13 and S14 (Supporting Information), to assess the rate capability and cycling stability, galvanostatic cycling tests were conducted at 0.5 C. The capacity retention and cycle stability of M_x_M″_3‐x_(HHTP)_2_ modified battery are better than those of PP‐based battery after 100 cycles. In particular, the Ni_1.35_Co_1.65_(HHTP)_2_/PP battery exhibits a high discharge capacity of 618 mAh g^−1^ at 1C rate and a capacity decay rate of 0.08% after 500 cycles (Figure S15, Supporting Information). These enhancements are ascribed to the robust chemical anchoring of LiPSs by cMOFs, which effectively suppress the shuttle effect and promote rapid charge transfer at the electrode‐electrolyte interface. In addition, the monometallic and bimetallic M_x_M″_3‐x_ (HHTP) _2_ separators retain more than 50% of the initial capacity after 100 cycles, which is not ideal. The main reason is that although cMOF greatly promotes the conversion of polysulfides, a large number of insolubles such as Li_2_S/Li_2_S_2_ will be rapidly deposited on the pores and surfaces of MOFs, so that the subsequent LiPSs cannot be effectively adsorbed and converted. And the violent reaction and the huge deposition specific surface area will irreversibly consume the electrolyte, resulting in a sharp increase in the internal resistance of the battery, an increase in polarization, and a rapid decay in capacity. Although the capacity decay behavior indicates that further optimization is needed, the current results demonstrate the conceptual validity of the cMOF membrane in regulating ion transport and alleviating LiPSs migration. These findings suggest that combining cMOF design with additional strategies, such as electrolyte engineering or cathode enhancement, can further enhance long‐term cycle stability. In addition, the specific capacity of PP‐battery increases abnormally with the increase of cycle, which may be due to the interface layer with ion screening function formed by PP separator during the cycle. At the beginning of the cycle, these sediments may hinder ion transport, but as the cycle progresses, the sedimentary layer may preferentially block the shuttle of polysulfides, optimize the Li transport path, and improve the sulfur utilization rate. And long‐term cycle may gradually activate the initial unreacted sulfur, showing an increase in apparent capacity. At the same time, these unstable sedimentary layers also affect the coulomb efficiency, resulting in abnormal fluctuations in the coulomb efficiency of the first 30 cycles.

As shown in the Figure S16 (Supporting Information), the charge–discharge profiles of cells equipped with M_x_M'_3‐x_(HHTP)_2_‐modified separators at various current densities. At 0.1 C, the Ni_1.35_Co_1.65_(HHTP)_2_/PP ‐based battery delivers a high specific capacity of 1020 mAh g^−1^, and maintains 440 mAh g^−1^ at 1 C (Figure S16d, Supporting Information). Similarly, the Ni_0.89_Cu_2.11_(HHTP)_2_/PP cell exhibits 905 mAh g^−1^ at 0.1 C and 450 mAh g^−1^ at 1 C (Figure S16e, Supporting Information). As the current density increases, the discharge plateau gradually shortens in all cells, which is primarily attributed to the intrinsically low conductivity of sulfur and LiPSs, as well as the limited diffusion rate of Li^+^ at high rates. Notably, the PP‐based cell exhibits a pronounced decrease in discharge plateau under high‐rate conditions, due to severe polysulfide shuttle and irreversible capacity loss. In contrast, both Ni_1.35_Co_1.65_(HHTP)_2_/PP and Ni_0.89_Cu_2.11_(HHTP)_2_/PP maintain well‐defined discharge plateaus even at 1 C, highlighting their ability to facilitate efficient redox kinetics and suppress the shuttle effect during high‐rate cycling.

**Figure 5:**
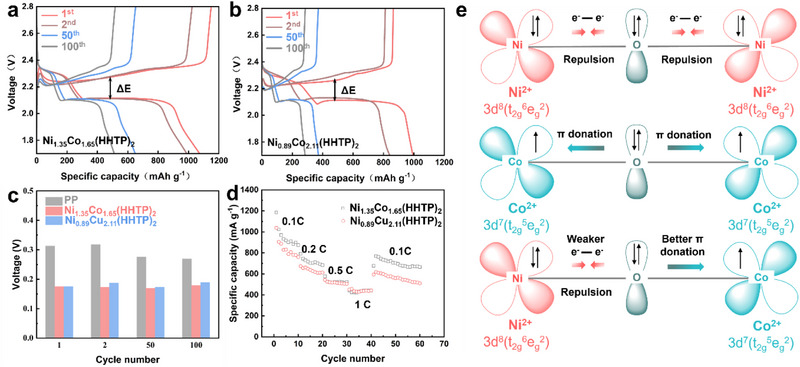
GCD profiles of a) Ni_1.35_Co_1.65_(HHTP)_2_ and b) Ni_0.89_Cu_2.11_(HHTP)_2_ modified separator battery. c) Voltage gap between the charge/discharge platform of the LSBs with different separators at 0.1 C. d) Rate performance of LSBs with different separators at different rates. e) Schematic representation of the electronic coupling between Co and Ni in Ni_1.35_Co_1.65_(HHTP)_2_.

By calculating the voltage hysteresis (ΔE) from the GCD curves (**Figure** **5**a,b; Figure S17, Supporting Information), it was observed that the Ni_1.35_Co_1.65_(HHTP)_2_/PP‐modified battery consistently maintains the smallest ΔE over 100 cycles (Figure 5c). This lower voltage hysteresis indicates improved reaction kinetics and reduced polarization during cycling. In addition, the rate capability of LSBs using different separators was assessed at different current densities (0.1, 0.2, 0.5, and 1 C), as shown in Figure 5d and Figure S18 (Supporting Information). The Ni_1.35_Co_1.65_(HHTP)_2_/PP‐based cell delivers a remarkable specific capacity of 1185 mAh g^−1^ at 0.1 C, and retains 440 mAh g^−1^ even at 1 C, demonstrating excellent high‐rate performance. In contrast, the Ni_0.89_Cu_2.11_(HHTP)_2_/PP cell shows comparatively lower capacities under the same conditions. The superior electrochemical behavior of Ni_1.35_Co_1.65_(HHTP)_2_ over Ni_0.89_Cu_2.11_(HHTP)_2_ can be fundamentally attributed to differences in their crystal field environments and d‐orbital electron configurations (Figure 5e), which influence their electronic conductivity, redox activity, and overall catalytic efficiency in Li_2_S_n_ conversion. Within the octahedral coordination environment provided by the HHTP ligand, Ni^2+^ (3d^8^, low‐spin) and Co^2+^ (3d⁷, high‐spin) ions exhibit favorable orbital alignment. The partially filled e_g_ orbitals of both cations enable synergistic π‐d orbital interactions through the bridging oxygen atoms of the HHTP framework.^[^ ^30^ ^]^ This results in a highly delocalized electronic structure, promoting fast electron redistribution and strong Li_2_S_n_ adsorption, particularly toward long‐chain species such as Li_2_S_6_ and Li_2_S_4_. The complementary electron donation and acceptance capacities of Ni^2+^ and Co^2+^ facilitate efficient redox catalysis and lower the energy barrier for Li_2_S_n_ conversion. Conversely, in Ni_0.89_Cu_2.11_(HHTP)_2_, the Cu^2+^ ion (3d^9^) is subject to Jahn‐Teller distortion, which leads to asymmetric elongation in its octahedral coordination as shown in the Figure S19 (Supporting Information).^[^ ^31^ ^]^ This distortion not only destabilizes the crystal field symmetry but also results in saturated e_g_ orbitals, limiting their ability to engage in effective orbital overlap or electron exchange with neighboring metal centers. Consequently, the electron delocalization is suppressed, the charge transfer dynamics are hindered, and the interaction with LiPSs is significantly weakened. The disparity in orbital occupation and distortion‐induced localization collectively explain the inferior electrocatalytic performance of Ni_0.89_Cu_2.11_(HHTP)_2_ relative to its Ni‐Co counterpart. These findings underscore the critical importance of crystal field matching and orbital synergy in the design of bimetallic MOF‐based materials for high‐performance LSB systems.^[32]^

Based on the above results, the battery with Ni_1.35_Co_1.65_(HHTP)_2_/PP separator shows outstanding electrochemical performance. To further verify the role of the separator, the chemical composition and microstructure of the separators after 100 charge–discharge cycles were analyzed using SEM and elemental mapping, as shown in Figure S20 (Supporting Information). In the PP and Super‐P/PP separators, a uniform oxygen (O) signal is observed, which is attributed to air exposure during SEM sample preparation. In contrast, the strong O signal in the Ni_1.35_Co_1.65_(HHTP)_2_/PP separator originates from the MOF coating itself. For the PP separator, a strong sulfur (S) signal is detected, and numerous Li_2_S_n_ deposits are observed on the surface (Figure S20a, Supporting Information), indicating severe polysulfide shuttle. Similarly, the Super‐P/PP separator shows a strong S signal in the Super‐P layer (Figure S20b, Supporting Information), suggesting that Super‐P fails to effectively suppress Li_2_S_n_ migration. In comparison, the Ni_1.35_Co_1.65_(HHTP)_2_/PP separator exhibits a strong and uniform S signal confined within the MOF layer, while the underlying PP layer shows a significantly weaker S signal (Figure S20c, Supporting Information). This distribution indicates that the Ni_1.35_Co_1.65_(HHTP)_2_/PP layer effectively captures migrating Li_2_S_n_ and prevents their diffusion toward the lithium anode. Additionally, a uniform carbon (C) signal is observed throughout the MOF layer, implying good electronic conductivity. This conductive matrix facilitates electron transfer, enabling reactivation of trapped sulfur species and mitigating irreversible deposition of Li_2_S/Li_2_S_2_.

## 3 Conclusion

In summary, we have successfully designed and fabricated improved separators modified by highly conjugated MOF material M_x_M'_3‐x_(HHTP)_2_ (M = Ni^2+^, Co^2+^, Cu^2+^). The modified separators can effectively inhibit the shuttle effect of Li_2_S_n_ and facilitate Li⁺ transport, thus significantly improving the comprehensive electrochemical performance of LSBs. In particular, benefiting from the synergistic interaction between nickel and cobalt metal centers, the bimetallic framework Ni_1.35_Co_1.65_(HHTP)_2_ exhibits an optimized pore structure, enhanced electrical conductivity, and a stronger chemical affinity for Li_2_S_n_ compared to the other counterparts. Therefore, the LSBs equipped with Ni_1.35_Co_1.65_(HHTP)_2_/PP achieves a high initial discharge capacity of 1148 mAh g^−1^ at 0.1 C and the capacity decay rate is 0.08% after 500 cycles at 1C. In general, this work provides new insights into the rational design of cMOF‐based separators and paves the way for the development of next‐generation high‐performance LSBs.

## Conflict of Interest

The authors declare no conflict of interest.

## Supporting information



Supporting Information

## Data Availability

The data that support the findings of this study are available in the supplementary material of this article.
