# Supplementary material for: Synergistic Ni‐Co Metal Nodes in a Conjugated MOF‐Modified Separator for High‐Performance Lithium‐Sulfur Batteries
Source: Adv Sci (Weinh). 2025 Sep 9;12(45):e13282. doi: 10.1002/advs.202513282 (PMC12677593; doi:10.1002/advs.202513282)
Supplement: Supplementary file 1 — Supporting Information [file ADVS-12-e13282-s001.docx]

**Supporting Information**

**Synergistic Ni-Co Metal Nodes in a Conjugated MOF**-**Modified Separator for** **High-Performance Lithium-Sulfur Batteries**

*Yuanhang Xu, Yuxuan Jiang, Xiang Yu, Yijing Gu, Mohsen Shakouri, Rongmei Zhu* and Huan Pang**

Y. Xu, Y. Jiang, X. Yu, Y. Gu, R. Zhu, and H. Pang

School of Chemistry and Chemical Engineering, Yangzhou University, Yangzhou, Jiangsu, 225009, P. R. China

E-mail: [rmzhu@yzu.edu.cn](mailto:rmzhu@yzu.edu.cn); huanpangchem@hotmail.com; [panghuan@yzu.edu.cn](mailto:panghuan@yzu.edu.cn)

X. Yu

School of Chemistry and Chemical Engineering, Chongqing University of Science and Technology, Chongqing, 401331, P. R. China

Y. Gu

School of Environmental Science, Nanjing Xiaozhuang University, Nanjing, Jiangsu, 211171, P. R. China

M. Shakouri

Canadian Light Source, University of Saskatchewan, Saskatoon, Saskatchewan, S7N 2V3, Canada

**Content**

[**Chemicals and synthesis** 4](#_Toc206884203)

[**Materials Characterization** 5](#_Toc206884204)

[**LiPSs adsorption test** 5](#_Toc206884205)

[**Li-S cell assembling and test** 6](#_Toc206884206)

[**Limitation** 6](#_Toc206884207)

[**Table S1.** ICP test of bimetallic samples. 7](#_Toc206884208)

[**Figure S1.** The structure of the simulated M_x_M'_3-x_(HHTP)_2_ nanorods were based on the structures shown in (a) and (b). 8](#_Toc206884209)

[**Figure S2.** TEM images of as-prepared (a) Ni_3_(HHTP)_2_ nanorods, (b) Co_3_(HHTP)_2_ nanorods, (c) Cu_3_(HHTP)_2_ nanorods, (d) Ni_1.35_Co_1.65_(HHTP)_2_ nanorods, (e) Ni_0.89_Cu_2.11_(HHTP)_2_ nanorods, (f) Co_0.92_Cu_2.08_(HHTP)_2_ nanorods. 9](#_Toc206884210)

[**Figure S3.** XRD images of the M_x_M'_3-x_(HHTP)_2_.^[1, 2]^ 10](#_Toc206884211)

[**Figure S4.** FT-IR patterns of the M_x_M'_3-x_(HHTP)_2_. 11](#_Toc206884212)

[**Figure S5.** N_2_ adsorption-desorption isotherms and pore size distribution of Ni_1.35_Co_1.65_(HHTP)_2_. 12](#_Toc206884213)

[**Figure S6.** N_2_ adsorption–desorption isotherms and pore size distribution of Ni_0.89_Cu_2.11_(HHTP)_2_. 13](#_Toc206884214)

[**Figure S7.** Optical photos of M_x_M'_3-x_(HHTP)_2_ adsorbed polysulfides changing with time. 14](#_Toc206884215)

[**Figure S8.** Solution before and after in-situ UV-Vis test. 15](#_Toc206884216)

[**Figure S9.** Li_2_S_4_ permeation in H-type cells based on PP, Ni_1.35_Co_1.65_(HHTP)_2_/PP and Ni_0.89_Cu_2.11_(HHTP)_2_/PP separators. 16](#_Toc206884217)

[**Figure S10.** The CV curves of LSB with different separators at different scanning rates, (a) Ni_3_(HHTP)_2_/PP, (b) Co_3_(HHTP)_2_/PP, (c) Cu_3_(HHTP)_2_/PP and (d) Co_0.92_Cu_2.08_(HHTP)_2_/PP. 17](#_Toc206884218)

[**Figure S11.** Diffusion coefficient of Li^+^ of LSBs with different separators. 18](#_Toc206884219)

[**Figure S12.** Nyquist plots of batteries with different separator. and the fitting circuit diagram of the Nyquist curve. 19](#_Toc206884220)

[**Figure S13.** Cycling performance of different separators at 0.5 C rate. 20](#_Toc206884221)

[**Figure S14.** Cycling performance of different separators at 0.5 C rate. 21](#_Toc206884222)

[**Figure S15.** Cycling performance of different separators at 1 C rate. 22](#_Toc206884223)

[**Figure S16.** GCD profiles of batteries with M_x_M'_3-x_(HHTP)_2_ modified separators. 23](#_Toc206884224)

[**Figure S17.** GCD curves of LSB with PP separator. 24](#_Toc206884225)

[**Figure S18.** Rate performance of LSBs with different separators at different rates. 25](#_Toc206884226)

[**Figure S19.** Jahn-Teller distortion of an octahedral molecule (Oh) with degenerate electronic states (middle), involves changes in bond lengths, leading to a JT hexagonal elongation (left) or –compression (right) geometry with a nondegenerate ground state. 26](#_Toc206884227)

[**Figure S20.** Cross-section SEM images and elemental maps of the (a) PP, (b) Super-P/PP, and (c) Ni_1.35_Co_1.65_(HHTP)_2_/PP separators after 100 cycles. 27](#_Toc206884228)

[**Figure S21.** TGA curve of the Super P/S 28](#_Toc206884229)

[**Table S2.** Comparisons of M_x_M'_3-x_(HHTP)_2_-modified separator with previous MOF-modified separators in LSBs. 29](#_Toc206884230)

[**References** 30](#_Toc206884231)

# Chemicals and synthesis

*Synthesis of Ni_3_(HHTP)_2_*: First, 7 mg HHTP and 10 mg Ni(CH_3_COO)_2_·4H_2_O were dispersed in glass bottle containing 4 mL of deionized water and ultrasound was performed until the mixture was uniformly dispersed. The mixture was then heated in an oven at 85 °C for 12 h. Finally, after the temperature of the mixture was naturally resumed to room temperature, it was washed with deionized water and acetone, centrifuged for three times, and the product was dried at room temperature overnight to obtain the sample.

*Synthesis of Co_3_(HHTP)_2_*: First, 7 mg HHTP and 10 mg Co(CH_3_COO)_2_·4H_2_O were dispersed in glass bottle containing 4 mL of deionized water and ultrasound was performed until the mixture was uniformly dispersed. The mixture was then heated in an oven at 85 °C for 12 h. Finally, after the temperature of the mixture was naturally resumed to room temperature, it was washed with deionized water and acetone, centrifuged for three times, and the product was dried at room temperature overnight to obtain the sample.

*Synthesis of Cu_3_(HHTP)_2_*: First, 7 mg HHTP and 11 mg Co(CH_3_COO)_2_·4H_2_O were dispersed in glass bottle containing 4 mL of deionized water and ultrasound was performed until the mixture was uniformly dispersed. The mixture was then heated in an oven at 85 °C for 12 h. Finally, after the temperature of the mixture was naturally resumed to room temperature, it was washed with deionized water and acetone, centrifuged for three times, and the product was dried at room temperature overnight to obtain the sample.

*Synthesis of Ni_x_Co_3-x_(HHTP)_2_*: First, 7 mg HHTP and 5 mg Ni(CH_3_COO)_2_·4H_2_O and 5 mg Co(CH_3_COO)_2_·4H_2_O were dispersed in glass bottle containing 4 mL of deionized water and ultrasound was performed until the mixture was uniformly dispersed. The mixture was then heated in an oven at 85 °C for 12 h. Finally, after the temperature of the mixture was naturally resumed to room temperature, it was washed with deionized water and acetone, centrifuged for three times, and the product was dried at room temperature overnight to obtain the sample.

*Synthesis of Ni_x_Cu_3-x_(HHTP)_2_*: First, 7 mg HHTP and 4.8 mg Ni(CH_3_COO)_2_·4H_2_O and 5.2 mg Cu(CH_3_COO)_2_·4H_2_O were dispersed in glass bottle containing 4 mL of deionized water and ultrasound was performed until the mixture was uniformly dispersed. The mixture was then heated in an oven at 85 °C for 12 h. Finally, after the temperature of the mixture was naturally resumed to room temperature, it was washed with deionized water and acetone, centrifuged for three times, and the product was dried at room temperature overnight to obtain the sample.

*Synthesis of Co_x_Cu_3-x_(HHTP)_2_*: First, 7 mg HHTP and 4.8 mg Co(CH_3_COO)_2_·4H_2_O and 5.2 mg Cu(CH_3_COO)_2_·4H_2_O were dispersed in glass bottle containing 4 mL of deionized water and ultrasound was performed until the mixture was uniformly dispersed. The mixture was then heated in an oven at 85 °C for 12 h. Finally, after the temperature of the mixture was naturally resumed to room temperature, it was washed with deionized water and acetone, centrifuged for three times, and the product was dried at room temperature overnight to obtain the sample.

*Preparation of cathode*: Super P/S composite was first prepared to be used as the activated material of LSB through melt-diffusion method (Super P and sublimation of sulfur mixed uniformly into the hydrothermal reaction kettle sealed, warmed to 155 ℃ insulation 12h). For the preparation of cathode, Super P/S, Super P, and N-methyl-2-pyrrolidone (NMP) solution containing 5 wt.% polyvinylidene fluoride (PVDF) (7:2:1 by weight) were milled together to form uniform slurry. Then, the slurry was coated on the carbon coated Al foil and dried in vacuum at 40 ℃ for 12 h. The obtained working electrodes were cut to circular electrode with a diameter of 12 mm. The mass loading of active sulfur was about 1.1-1.3 mg cm^−2^

*Preparation of modified separators*: 42 mg of M_x_M'_3-x_(HHTP)_2_ powder and 6 mg of Super P were weighed and ground until homogeneous, then 125 mg of pre-formulated NMP solution containing 5 wt.% PVDF was added and applied to the surface of the PP separator using a tetrahedral spatula (set to a thickness of 25 μm). The separators were dried in a vacuum oven at 40 °C for 12 h. Finally, the separators were cut into 16 mm pieces.

# Materials Characterization

The Powder X-ray diffraction (PXRD) patterns were performed by Bruker AXS D8 advance with Cu Kα radiation of 40 kV (λ=1.5418 Å). Scanning electron microscopy (SEM) images were obtained by Zeiss Supra 55 microscope. Transmission electron microscopy (TEM) and Energy Dispersive Spectrometer (EDS) elemental mapping scans were recorded using Tecnai G2 F30 S-TWIN at an acceleration voltage of 300 kV. Thermogravimetry analysis (TGA) was conducted with Diamond TG/DTA thermal analyzer (PerkinElmer, USA) to calculate the sulfur content of prepared samples. FT-IR measurement was investigated on a TENSOR27. X-ray photoelectron spectroscopy (XPS) analysis was carried out using a Thermo Scientific ESCALAB 250Xi X-ray photoelectron spectrometer with Al Kα radiation of 1486.6 eV as the excitation source. The survey thickness is 2-3 nm.

# LiPSs adsorption test

For the adsorption test, 30 mg M_x_M'_3-x_(HHTP)_2_ were soaked in 4 mL Li2S4 solution (10 mmol/L). The Li_2_S_4_ solution were prepared according to the reaction Equation S1:

3S + Li_2_S ↔ Li_2_S_4_ (S1)

Using a solvent mixture of 1,3-Dioxolane (DOL) and dimethoxyethane (DME) (1:1 in volume). UV-vis spectra of the above solutions (diluted 5 times before testing) were recorded by using a UV2550 instrument (Shimadzu, Japan). The concentration variations in these solutions were detected by the UV-vis spectroscopy.

# Li-S cell assembling and test

The CR 2032-type coin cells were fabricated using the working electrode, lithium foil as the counter and anode electrode, Celgard 2400 as the separator. The electrolyte was used 1.0 mol L^-1^ lithium bis(trifluoromethanesulfonyl)imide (LiTFSI Sigma-Aldrich (USA), 99.95%) in 1, 3-dioxolane (DOL, Sigma-Aldrich (USA), 99.0%) and 1,2-dimethoxyethane (DME, Sigma-Aldrich (USA), 99.0% (volume ratio, 1:1) with 1 wt.% LiNO_3_ in an argon-filled glove box (where both water and oxygen levels are below 0.1 ppm. The value of the electrolyte to S (E/S) ratio is 15 μL mg^-1^(according to the weight of S). The GCD tests were estimated in the voltage window of 1.7 - 2.8 V. The rate capability was also tested by varying the current density from 0.1 C to 1 C (1 C = 1675 mAh g^-1^) on a battery measurement system (CT2001A, Wuhan Land, China) at room temperature. CV and EIS curves were measured on an electrochemical workstation (DH7000C, Donghua, Jiangsu, China). CV curves were performed from 2.8 V to 1.7 V (vs Li^+^/Li) at a scanning rate of 0.1 mV s^-1^, and the frequency of EIS was performed form 100 kHz to 0.01 Hz at open-circuit potential.

# Limitation

In this work, the discussion of electronic orbitals is used only as a qualitative interpretation to support the experimental findings. Since no rigorous theoretical calculations were performed, the orbital analysis is based on simplified assumptions and previously reported concepts. Therefore, the conclusions regarding orbital contributions should be considered indicative trends rather than quantitative predictions. A more accurate understanding would require detailed theoretical studies, which are beyond the scope of the present work.

# Table S1. ICP test of bimetallic samples.

| Sample | Co (wt%) | Ni (wt%) | Cu (wt%) | Molar ratio (Co:Ni:Cu) |
| --- | --- | --- | --- | --- |
| Ni_1.35_Co_1.65_ (HHTP)_2_ | 54.9 | 45.1 | - | 1.65:1.35:0 |
| Ni_0.89_Cu_2.11_ (HHTP)_2_ | - | 29.7 | 70.3 | 0:0.93:2.07 |
| Co_0.92_Cu_2.08_ (HHTP)_2_ | 30.6 | - | 69.7 | 0.92:0:2.08 |


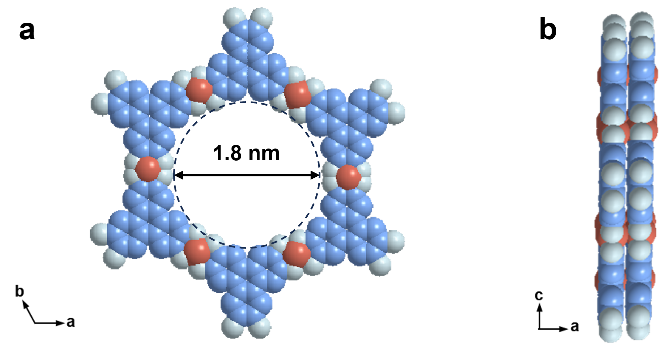


# **Figure S1.** The structure of the simulated M_x_M'_3-x_(HHTP)_2_ nanorods were based on the structures shown in (a) and (b).


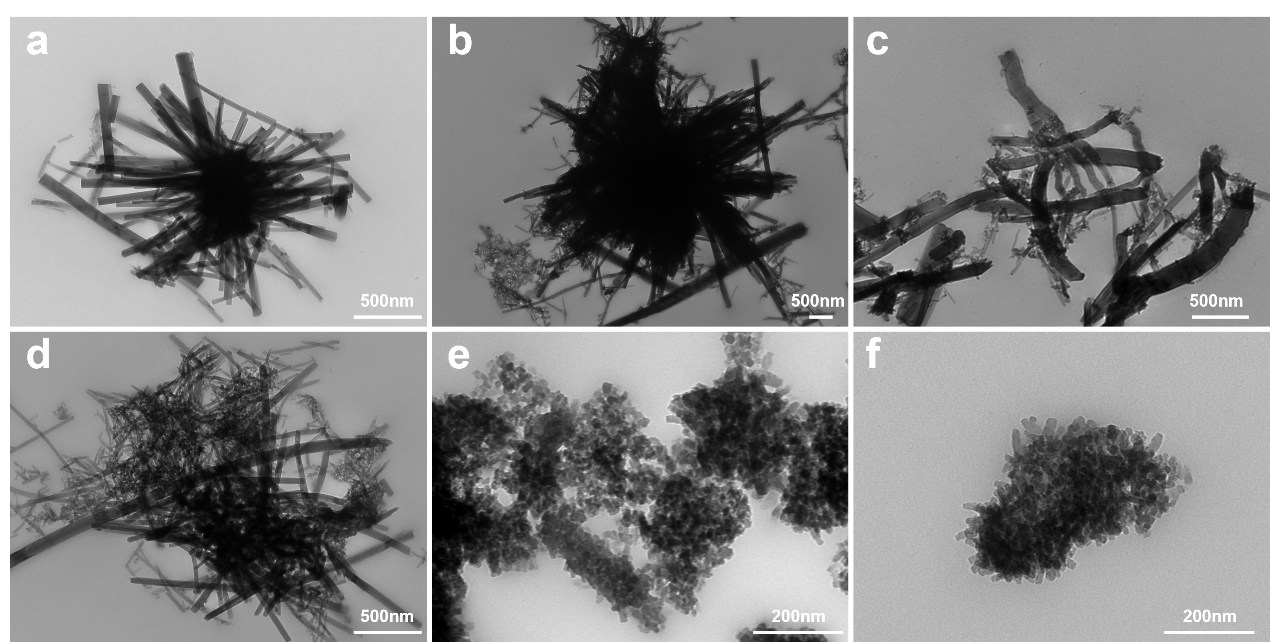


# **Figure S2.** TEM images of as-prepared (a) Ni_3_(HHTP)_2_ nanorods, (b) Co_3_(HHTP)_2_ nanorods, (c) Cu_3_(HHTP)_2_ nanorods, (d) Ni_1.35_Co_1.65_(HHTP)_2_ nanorods, (e) Ni_0.89_Cu_2.11_(HHTP)_2_ nanorods, (f) Co_0.92_Cu_2.08_(HHTP)_2_ nanorods.


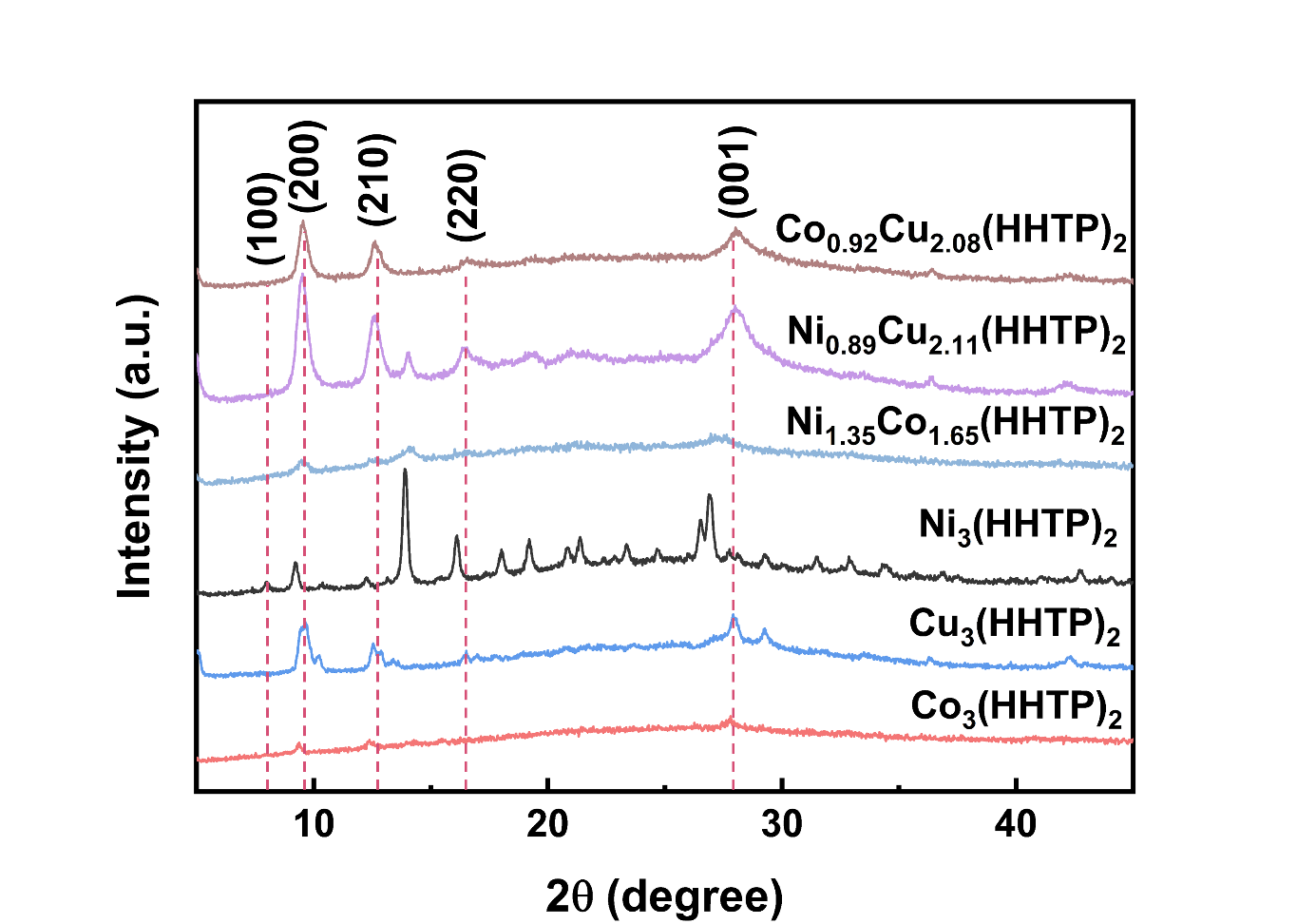


# **Figure S3.** XRD images of the M_x_M'_3-x_(HHTP)_2_.^[1, 2]^


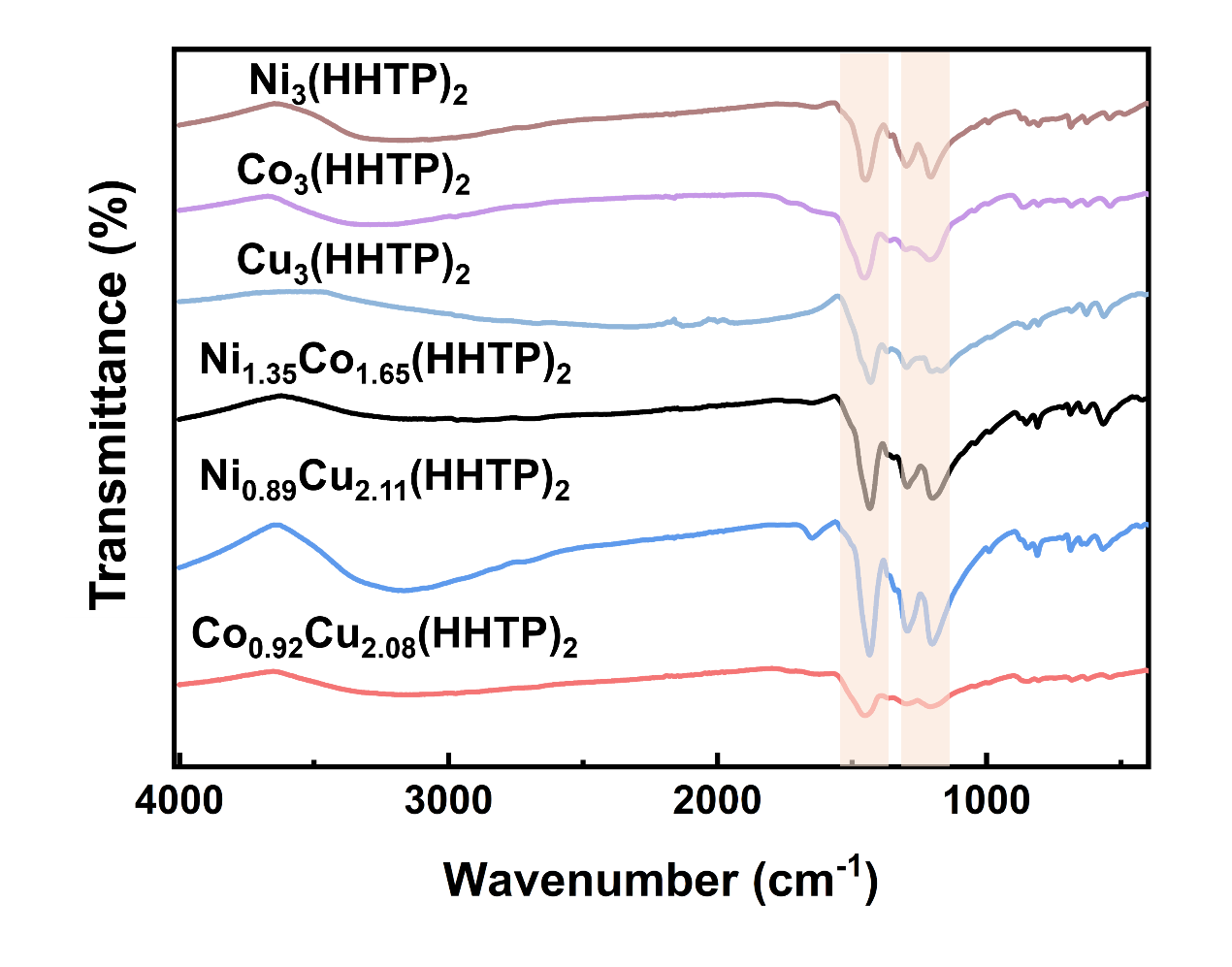


# **Figure S4.** FT-IR patterns of the M_x_M'_3-x_(HHTP)_2_.


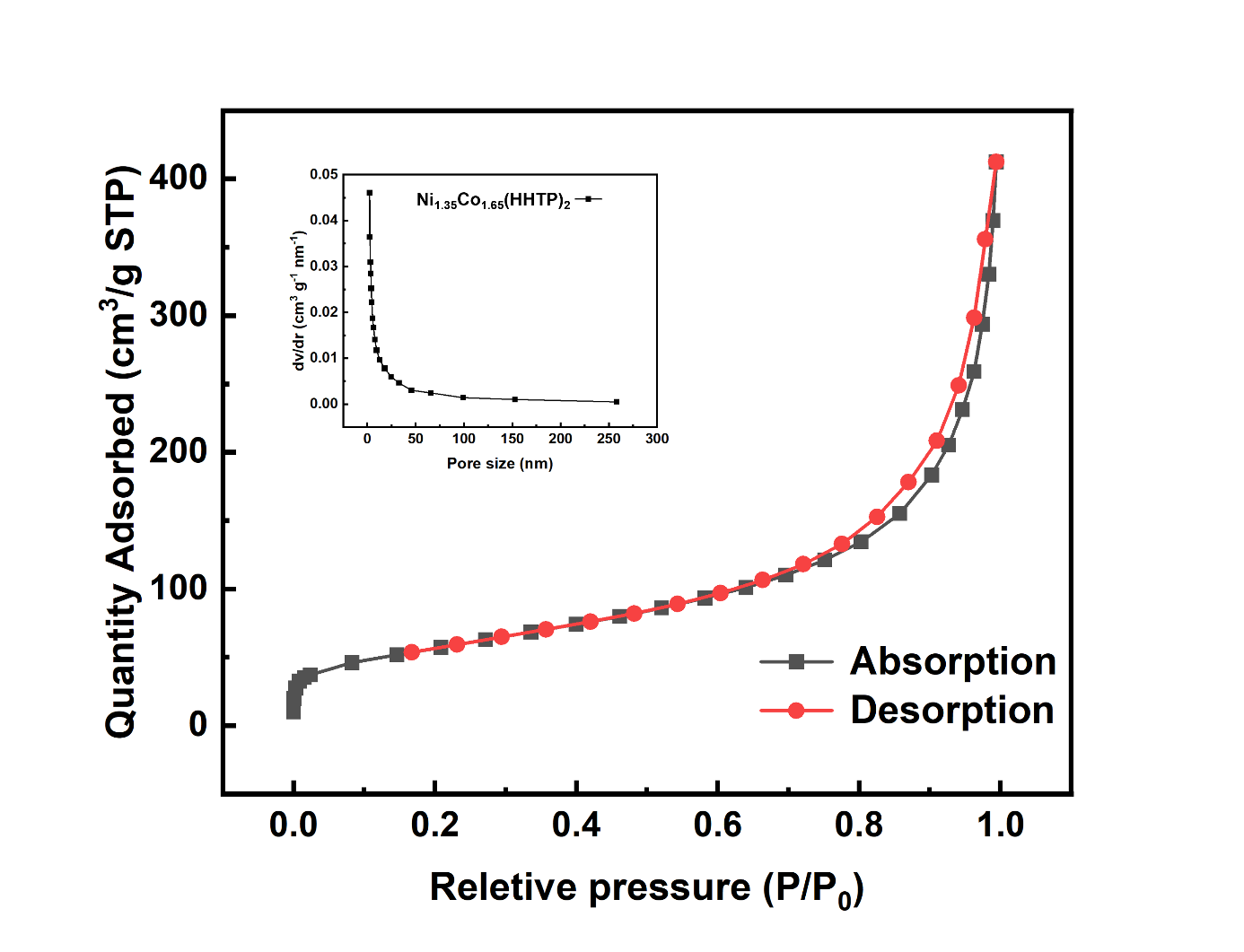


# Figure S5. N_2_ adsorption-desorption isotherms and pore size distribution of Ni_1.35_Co_1.65_(HHTP)_2_.


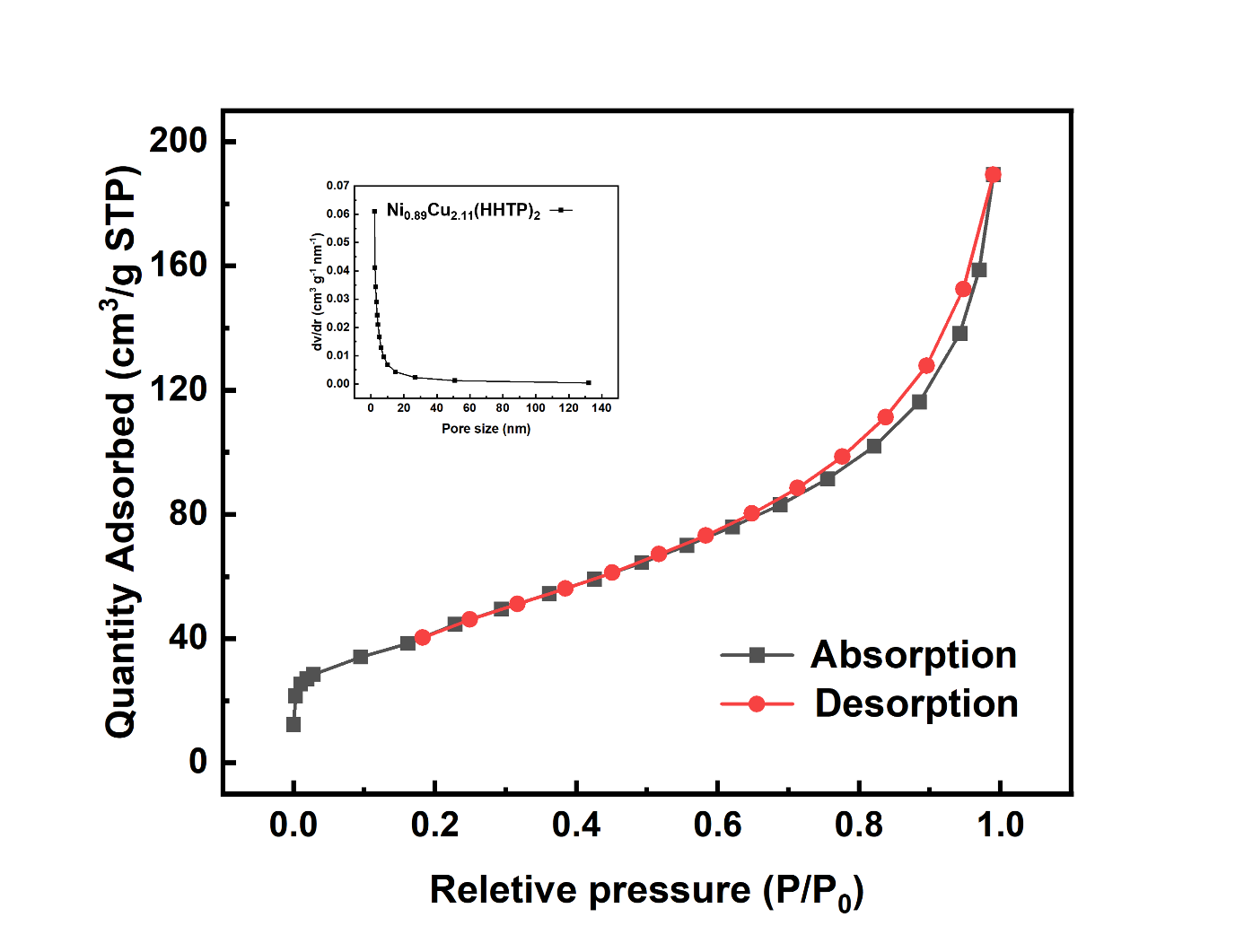


# Figure S6. N_2_ adsorption–desorption isotherms and pore size distribution of Ni_0.89_Cu_2.11_(HHTP)_2_.


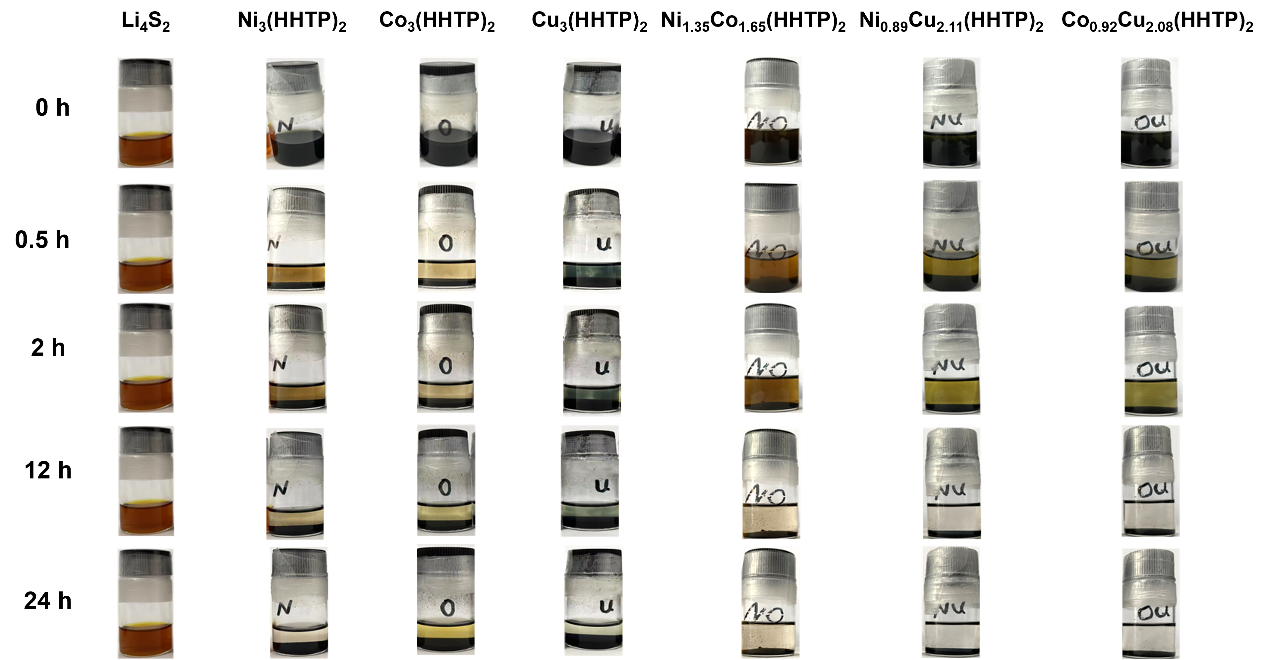


# **Figure S7**. Optical photos of M_x_M'_3-x_(HHTP)_2_ adsorbed polysulfides changing with time.

**
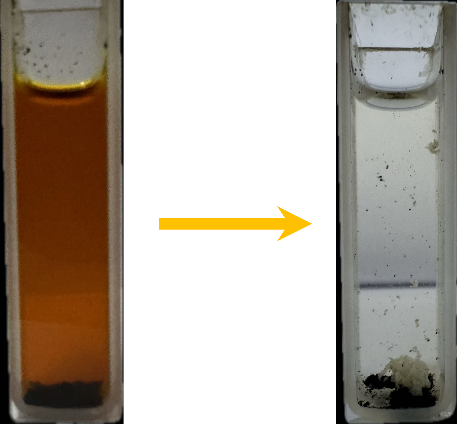
**

# Figure S8. Solution before and after in-situ UV-Vis test.


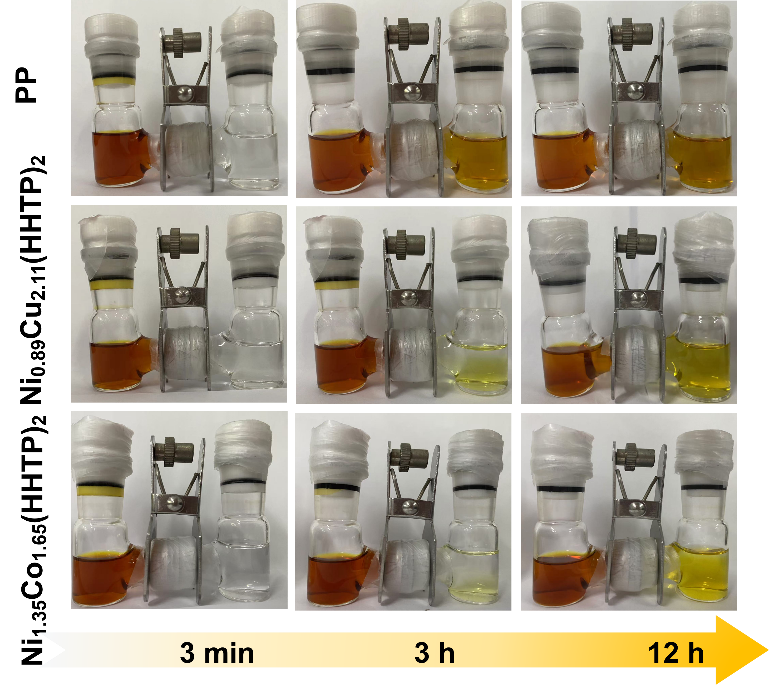


# **Figure S9**. Li_2_S_4_ permeation in H-type cells based on PP, Ni_1.35_Co_1.65_(HHTP)_2_/PP and Ni_0.89_Cu_2.11_(HHTP)_2_/PP separators.


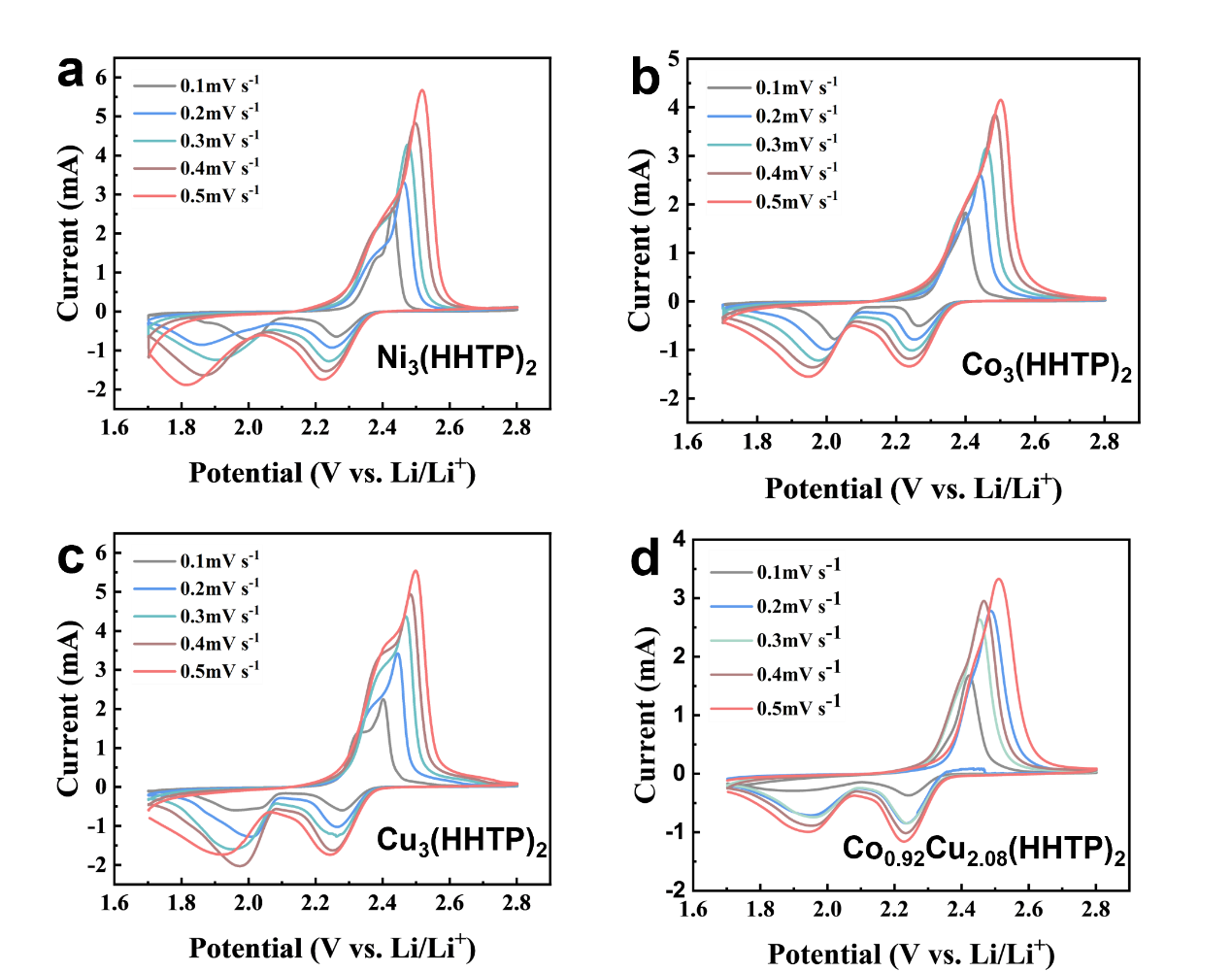


# **Figure S10**. The CV curves of LSB with different separators at different scanning rates, (a) Ni_3_(HHTP)_2_/PP, (b) Co_3_(HHTP)_2_/PP, (c) Cu_3_(HHTP)_2_/PP and (d) Co_0.92_Cu_2.08_(HHTP)_2_/PP.

**
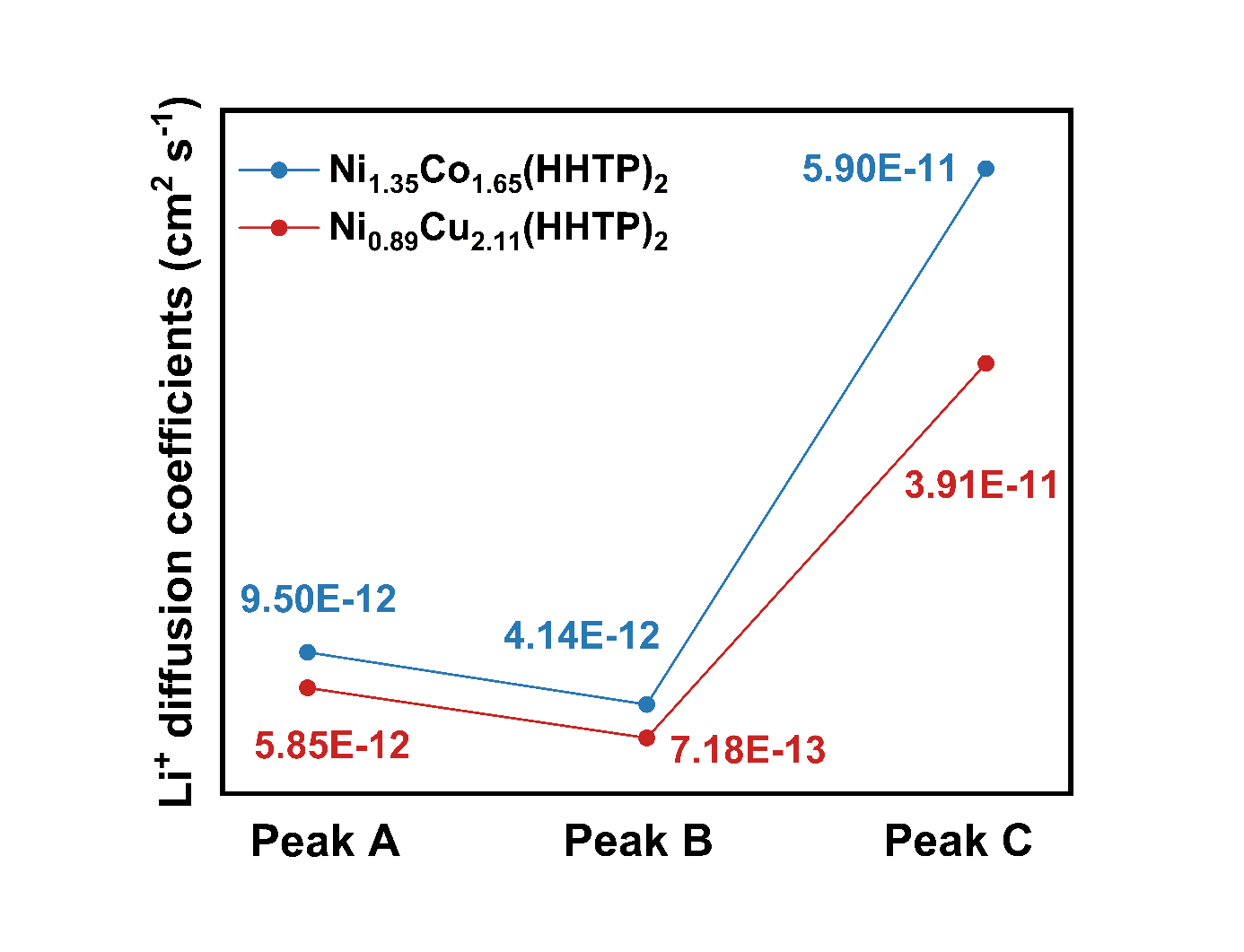
**

# Figure S11. Diffusion coefficient of Li^+^ of LSBs with different separators.


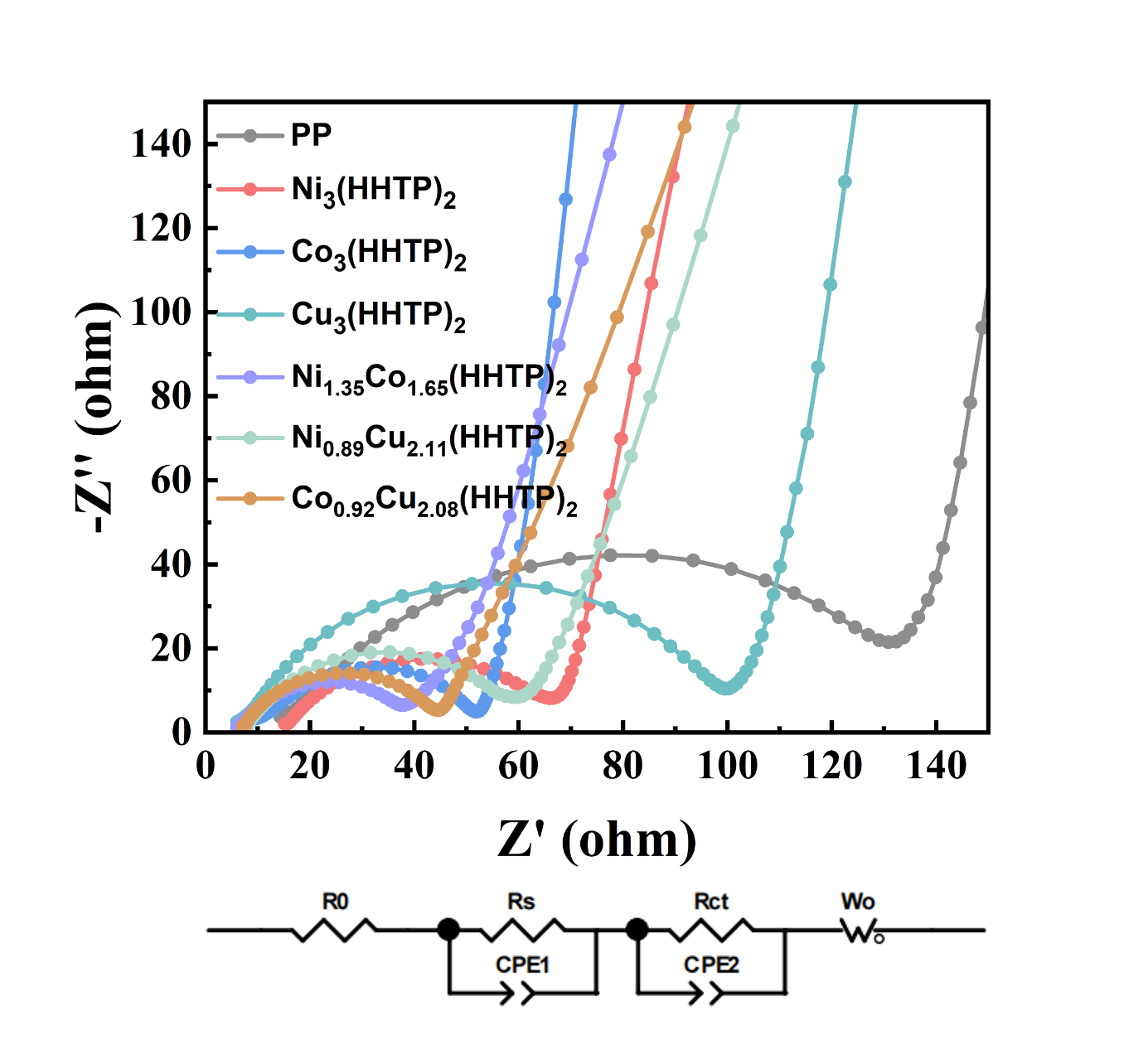


# **Figure S12**. Nyquist plots of batteries with different separator. and the fitting circuit diagram of the Nyquist curve.


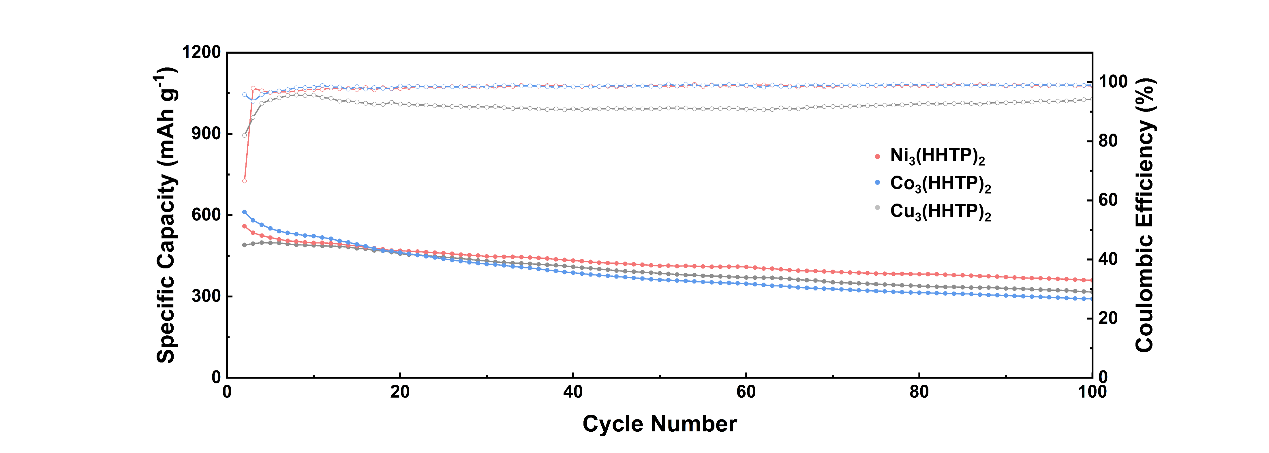


# **Figure S13**. Cycling performance of different separators at 0.5 C rate.


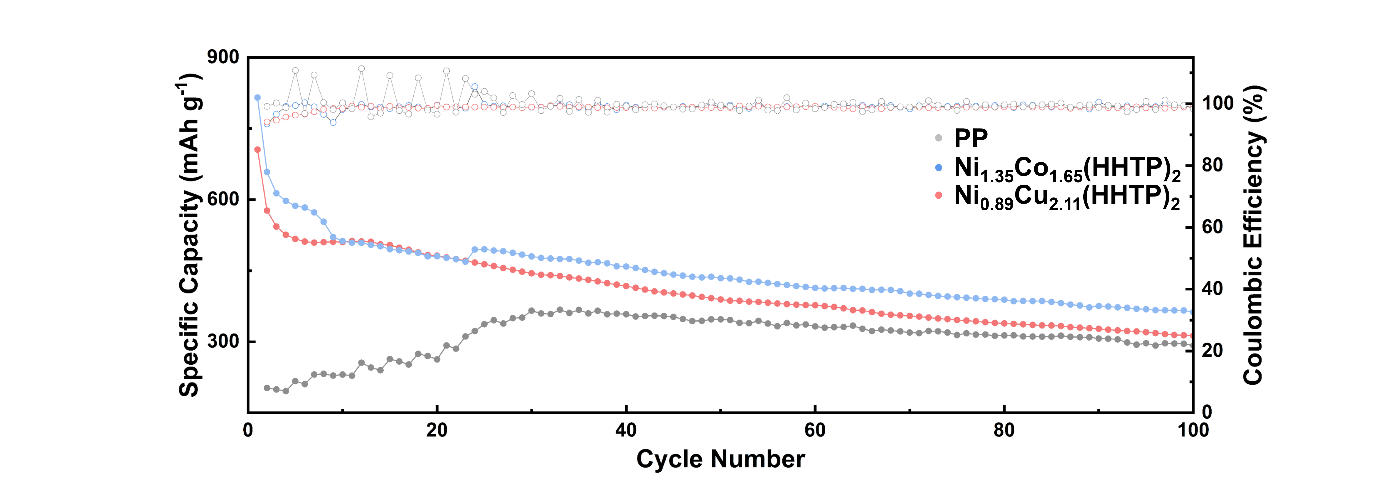


# Figure S14. Cycling performance of different separators at 0.5 C rate.





# Figure S15. Cycling performance of different separators at 1 C rate.


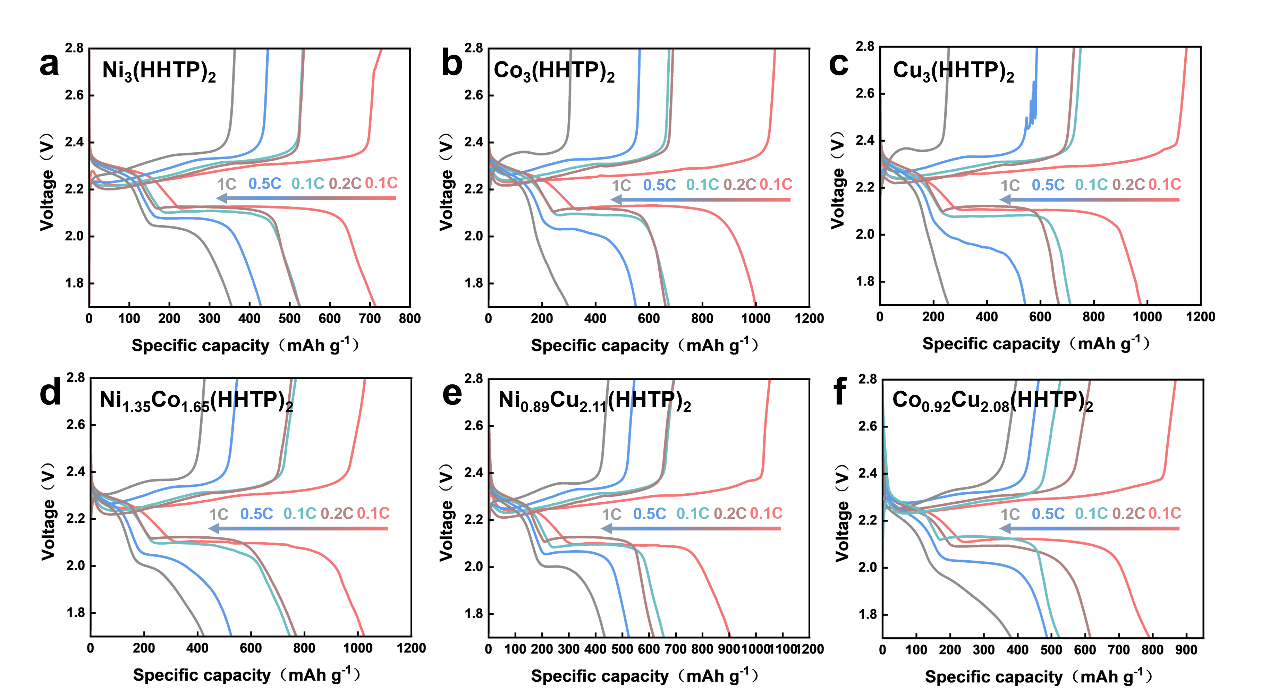


# **Figure S16**. GCD profiles of batteries with M_x_M'_3-x_(HHTP)_2_ modified separators.


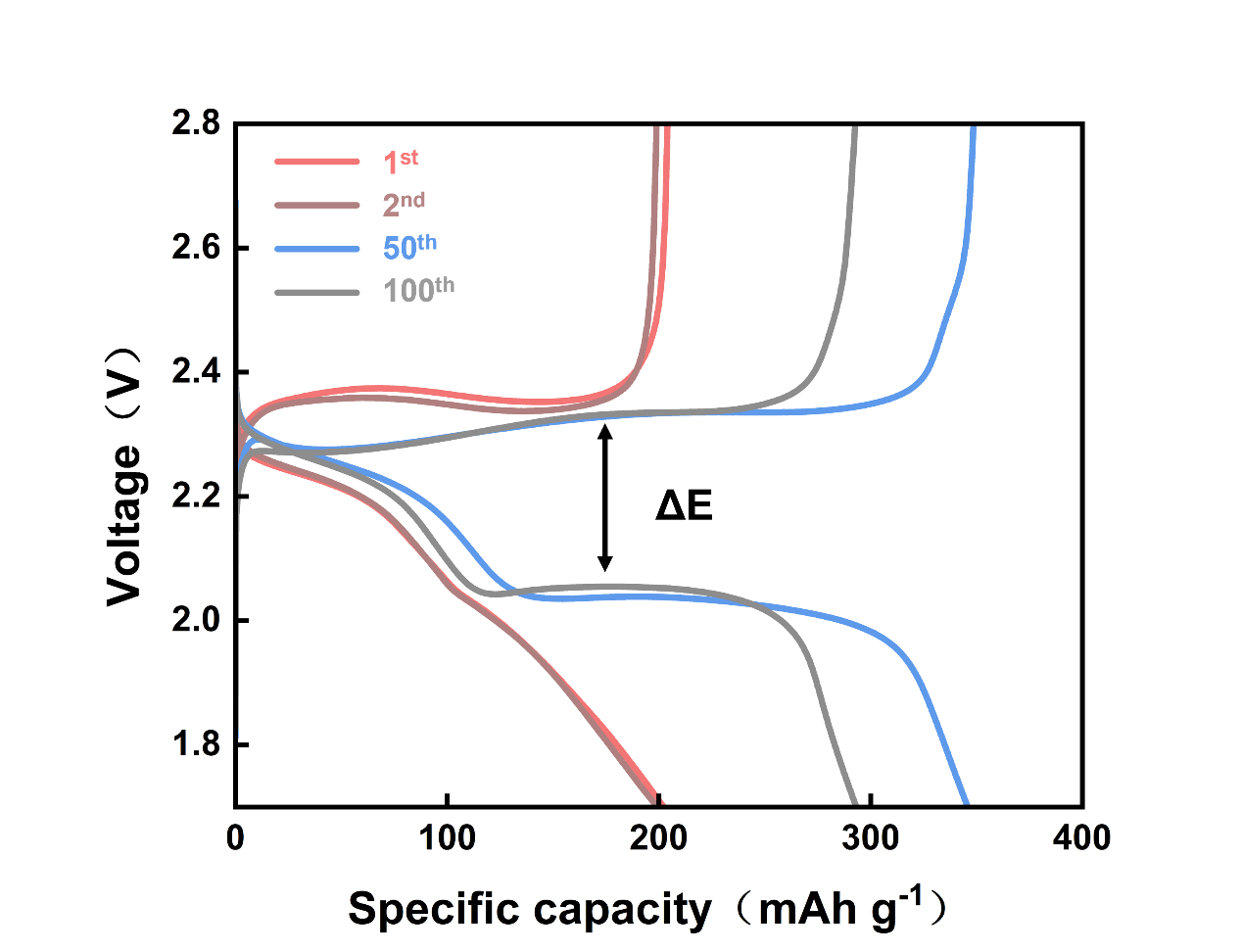


# Figure S17. GCD curves of LSB with PP separator.


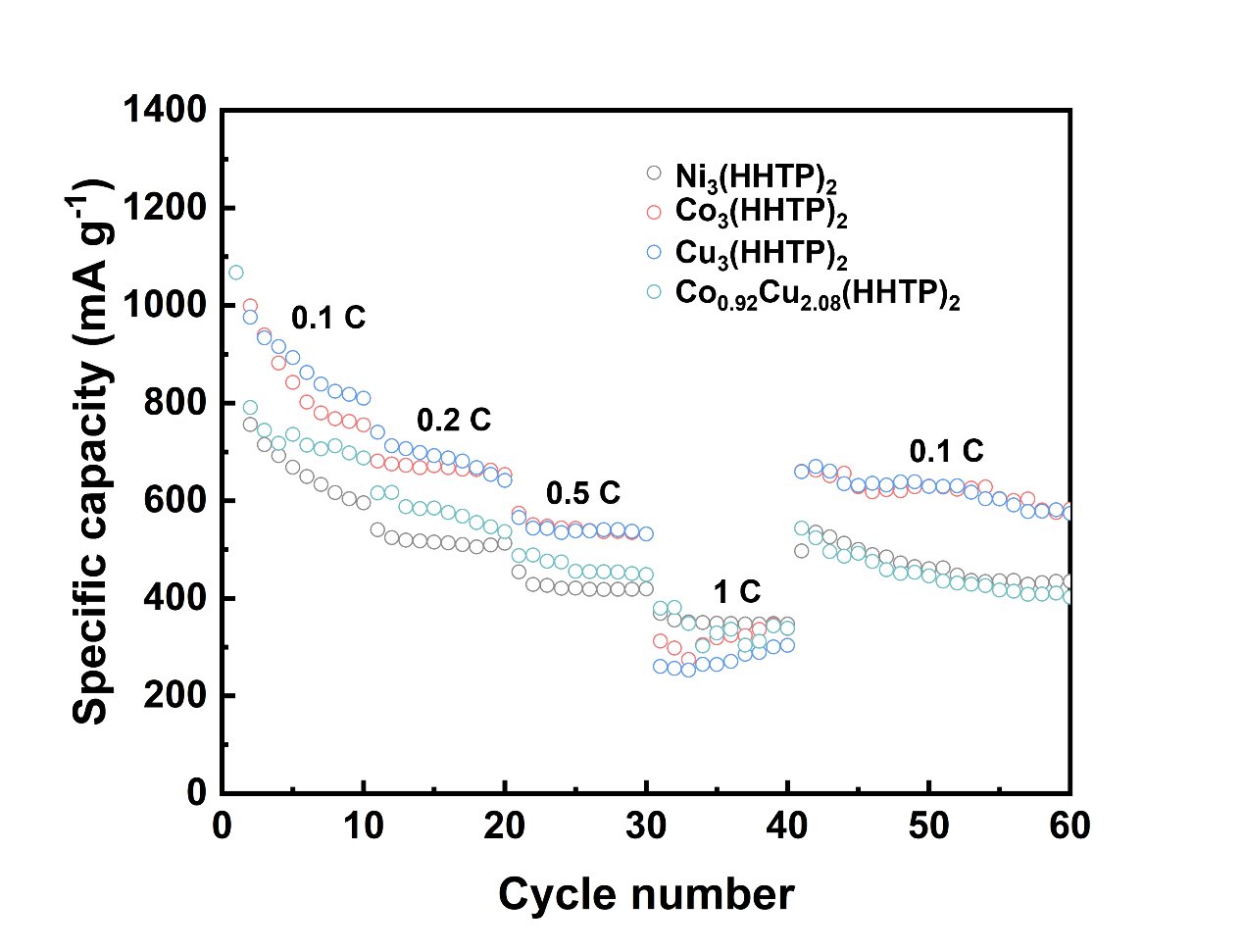


# **Figure S18**. Rate performance of LSBs with different separators at different rates.


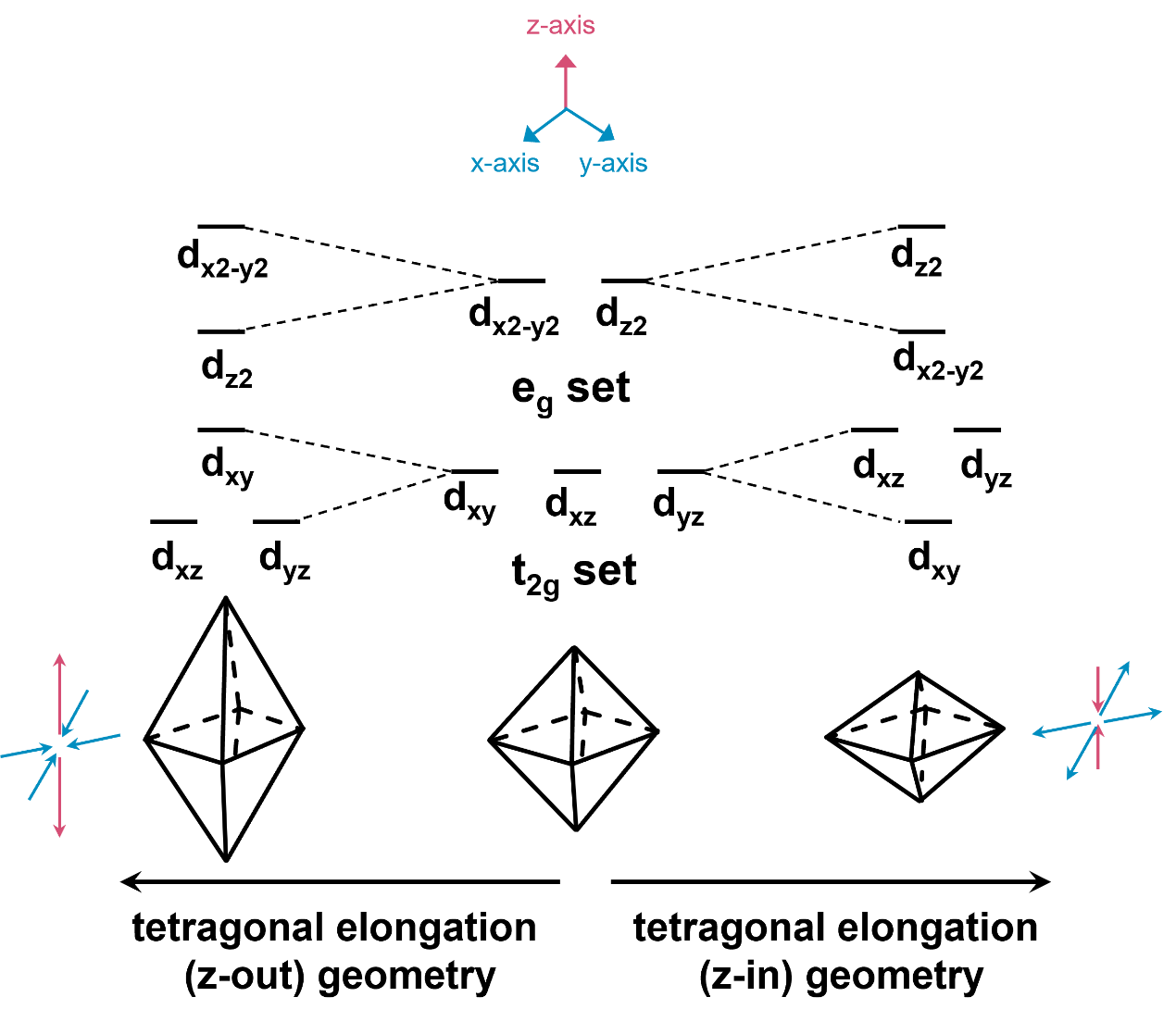


# Figure S19. Jahn-Teller distortion of an octahedral molecule (Oh) with degenerate electronic states (middle), involves changes in bond lengths, leading to a JT hexagonal elongation (left) or –compression (right) geometry with a nondegenerate ground state.


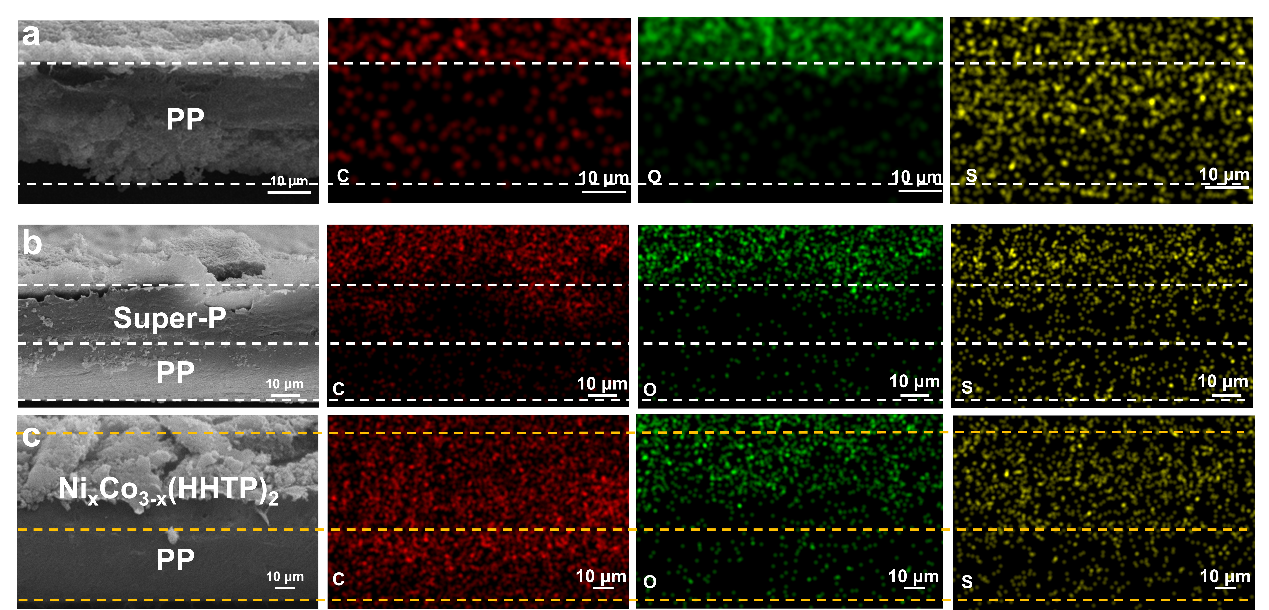


# Figure S20. Cross-section SEM images and elemental maps of the (a) PP, (b) Super-P/PP, and (c) Ni_1.35_Co_1.65_(HHTP)_2_/PP separators after 100 cycles.


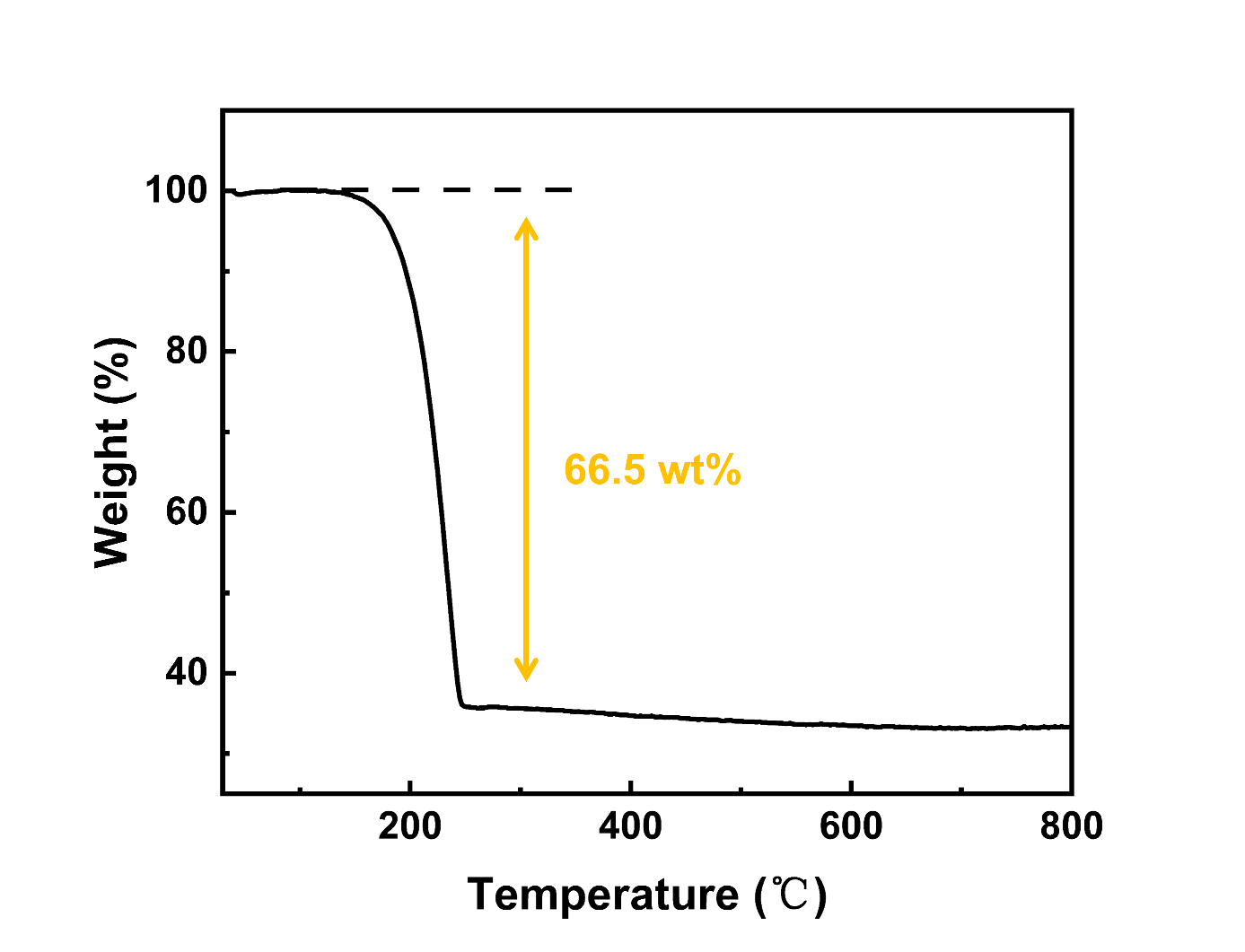


# Figure S21. TGA curve of the Super P/S

# Table S2. Comparisons of M_x_M'_3-x_(HHTP)_2_-modified separator with previous MOF-modified separators in LSBs.

| **Modified material** | **Sulfur in composite**  **[wt.%]** | **Initial discharge**  **capacity [mAh g^−1^]** | **Cycling performance**  **[mAh g^−1^]** | **References** |
| --- | --- | --- | --- | --- |
| FJU | 66 | 955.7 (0.5 C) | 493.8 (500 cycles, 1 C) | [3] |
| Ni_3_(HITP)_2_ | / | 1220.1 (0.1 C) | 585.4 (300 cycles, 0.5 C) | [4] |
| Ni-HAB@CNT | / | 1015 (0.1 C) | 1070 (200 cycles, 0.2 C) | [5] |
| 2D-Cu-BDC | 70 | 1231 (0.1 C) | 603 (500 cycles, 1 C) | [6] |
| ZIF-7 | 63 | 1025 (0.25 C) | 452 (300 cycles, 0.25 C) | [7] |
| ZIF-8 | 63 | 989 (0.25 C) | 403 (300 cycles, 0.25 C) | [7] |
| UiO-66 | 80 | 1147.4 (0.2 C) | 964.1 (200 cycles, 0.5 C) | [8] |
| 2D-NiCo MOF/CNT | / | 1132.7 (0.5 C) | 709.1 (300 cycles, 1 C) | [9] |
| HKUST-1 | 70 | 1072 (0.2 C) | 772.9 (200 cycles, 0.2 C) | [10] |
| Mn-BTC | / | 1450 (0.1 C) | 1073 (80 cycles, 0.1 C) | [11] |
| This work | 75 | 1148 (0.1 C) | 345 (500 cycles, 1 C) |  |

# References

[1] J. Li, Y. Huang, Y. Zhou, H. Dong, H. Wang, H. Shan, Y. Li, M. Xu, X. Wang, ACS Appl. Nano Mater. 6(24) (2023) 22916-22926.

[2] B. Geng, F. Yan, X. Zhang, Y. He, C. Zhu, S.L. Chou, X. Zhang, Y. Chen, Adv. Mater. 33(49) (2021).

[3] Y. Chen, L. Zhang, H. Pan, J. Zhang, S. Xiang, Z. Cheng, Z. Zhang, J. Mater. Chem. A 2021, 9, 26929.

[4] H. Chen, Y. Xiao, C. Chen, J. Yang, C. Gao, Y. Chen, J. Wu, Y. Shen, W. Zhang, S. Li, F. Huo, B. Zheng, ACS Appl. Mater. Interfaces 2019, 11, 11459.

[5] T. Guo, Y. Ding, C. Xu, W. Bai, S. Pan, M. Liu, M. Bi, J. Sun, X. Ouyang,X. Wang, Y. Fu, J. Zhu, Adv. Sci. 2023, 10, 2302518.

[6] Z. Chang, Y. Qiao, J. Wang, H. Deng, H. Zhou, J. Mater. Chem. A 2021, 9, 4870.

[7] M. Li, Y. Wan, J.-K. Huang, A. H. Assen, C.-E. Hsiung, H. Jiang, Y. Han, M. Eddaoudi, Z. Lai, J. Ming, L.-J. Li, ACS Energy Lett. 2017, 2, 2362.

[8] J. Han, S. Gao, R. Wang, K. Wang, M. Jiang, J. Yan, Q. Jin, K. Jiang, J. Mater. Chem. A 2020, 8, 6661.

[9] P. Feng, W. Hou, Z. Bai, Y. Bai, K. Sun, Z. Wang, Chin. Chem. Lett. 2023, 34, 107427.

[10] Bai, S.; Liu, X.; Zhu, K.; Wu, S.; Zhou, H. Metal–Organic Framework-Based Separator for Lithium–Sulfur Batteries. Nat. Energy 2016, 1, 16094–16099.

[11] Suriyakumar, S.; Kanagaraj, M.; Kathiresan, M.; Angulakshmi, N.; Thomas S.; Stephan, A. M. Electrochim. Acta 2018, 265, 151–159.
